# Intrathecal versus intravenous umbilical cord mesenchymal stem cells for ischemic stroke sequelae

**DOI:** 10.1093/stcltm/szaf063

**Published:** 2025-11-24

**Authors:** Liem Thanh Nguyen, Thuy Thi Ngoc Nguyen, Kien Trung Nguyen, Lam Nam Phung, Van Thanh Hoang, Trang Thi Kieu Phan, Minh Van Pham, Anh Thi Phuong Nguyen, Doan Van Ngo, Anh Van Nguyen, Chi Van Nguyen

**Affiliations:** Vinmec Research Institute of Stem Cell and Gene Technology, College of Health Sciences, VinUniversity, Vinhomes Ocean Park, Gia Lam District, Hanoi, Vietnam; Vinmec Times City International Hospital, Vinmec HealthCare System, 458 Minh Khai Street, Hai Ba Trung District, Hanoi, Vietnam; Ha Noi Medical University, 1 Ton That Tung, Dong Da District, Hanoi, Vietnam; Vinmec Research Institute of Stem Cell and Gene Technology, College of Health Sciences, VinUniversity, Vinhomes Ocean Park, Gia Lam District, Hanoi, Vietnam; Vinmec Times City International Hospital, Vinmec HealthCare System, 458 Minh Khai Street, Hai Ba Trung District, Hanoi, Vietnam; Vinmec Research Institute of Stem Cell and Gene Technology, College of Health Sciences, VinUniversity, Vinhomes Ocean Park, Gia Lam District, Hanoi, Vietnam; Vinmec Research Institute of Stem Cell and Gene Technology, College of Health Sciences, VinUniversity, Vinhomes Ocean Park, Gia Lam District, Hanoi, Vietnam; Ha Noi Medical University, 1 Ton That Tung, Dong Da District, Hanoi, Vietnam; Ha Noi Rehabilitation Hospital, 35 Le Van Thiem, Thanh Xuan District, Hanoi, Vietnam; Vinmec Times City International Hospital, Vinmec HealthCare System, 458 Minh Khai Street, Hai Ba Trung District, Hanoi, Vietnam; Vinmec Times City International Hospital, Vinmec HealthCare System, 458 Minh Khai Street, Hai Ba Trung District, Hanoi, Vietnam; Vinmec Times City International Hospital, Vinmec HealthCare System, 458 Minh Khai Street, Hai Ba Trung District, Hanoi, Vietnam; National Rehabilitation Hospital, 27 Le Loi, Sam Son, Thanh Hoa, Vietnam

**Keywords:** ischemic stroke, umbilical cord-derived mesenchymal stem cells, intravenous infusion, intrathecal infusion, neurological recovery

## Abstract

**Background:**

Stroke is a leading cause of death worldwide. Traditional treatments have limitations, stem cell therapy has potential for regeneration after ischemic stroke. This study evaluated the safety and efficacy of allogeneic umbilical cord-derived mesenchymal stem cell (UC-MSC) infusion via the intravenous (IV) and intrathecal (IT) routes for treating neurological sequelae after ischemic stroke.

**Methods:**

This phase II randomized controlled trial involved 32 patients aged 40–75 years with neurological sequelae after ischemic stroke. The patients were randomly assigned into two groups: 16 received two IT UC-MSC infusions plus rehabilitation therapy, and 16 received two IV UC-MSC infusions plus rehabilitation therapy. Additionally, 16 matched controls, paired with the IT group by sex, age (±5 years), and NIHSS, received only rehabilitation. UC-MSCs were administered at 1.5 × 10^6^ cells/kg at baseline and 3 months. Outcomes were assessed at baseline, 3, 6, and 12 months using NIHSS, FIM, MAS, FMS, and SF-36.

**Results:**

No severe adverse events related to UC-MSC therapy were observed. Adverse event rate was lower in the IV group than the IT group. At 6 months, the IV group demonstrated significant improvements in NIHSS (p = 0.046), FIM (p = 0.028), and SF-36 (p < 0.001). At 12 months, both UC-MSC groups showed significant improvements, with greater effects in the IV group (p < 0.001 for SF-36).

**Conclusion:**

Both IV and IT UC-MSC infusions improved neurological recovery and quality of life, with fewer adverse events in the IT group.

**Trial registration:**

NCT05292625

## Graphical abstract

**Graphical Abstract:**
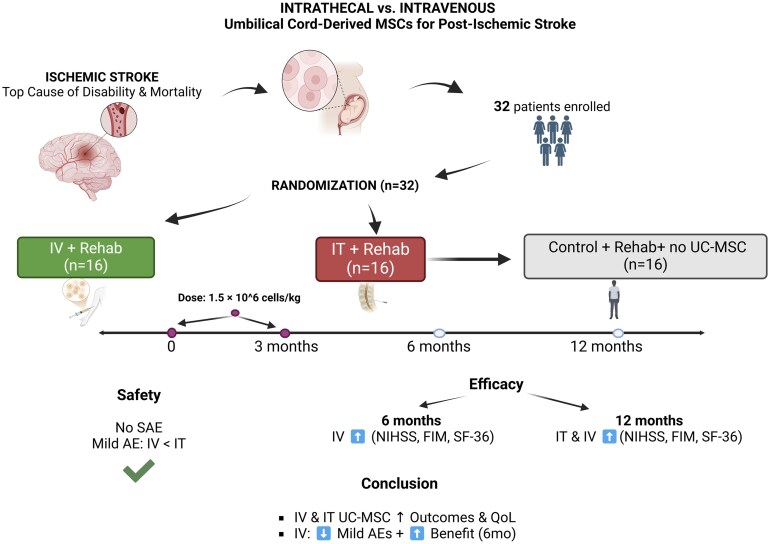

Lessons learned

- The improved efficacy and reduced adverse events observed in the IV group emphasize its promise as the preferred delivery method in general clinical practice.
- The absence of serious adverse events associated with UC-MSCs underscores the feasibility of cell-based therapy for stroke patients.
- Significant improvements observed at 12 months highlight the importance of extended follow-up to capture sustained benefits and inform long-term treatment strategies.
- Future studies should focus on optimizing cell dosage, infusion frequency, and patient selection to maximize therapeutic impact and open the door to larger-scale clinical trials.

Significance statement

This trial directly compared IV and IT infusions of UC-MSCs for stroke recovery. Both methods were well tolerated and led to improvements in neurological function. However, the IV route delivered greater clinical benefits with fewer adverse events. These results offer strong support for a promising cell-based treatment that may overcome some of the challenges of traditional post-stroke therapies. Moreover, the findings not only highlight the potential of UC-MSCs but also offer valuable insights for future research focused on improving treatment delivery, which could eventually lead to better care in stroke rehabilitation.

## Introduction

Stroke is a condition caused by an interruption in the blood supply to the brain, leading to localized brain cell death if the incidence lasts 24 hours or more^1^. Stroke is the second leading cause of death globally, and is the primary cause of long-term disability^2^. In 2013, approximately 6.5 million people died from stroke, and 33 million stroke survivors were living with neurologic sequelae^3^. Despite advancements in acute ischemic stroke management, including thrombolysis and mechanical thrombectomy, many patients suffer from persistent neurological deficits^4–6^. Traditional treatments have limited effectiveness in promoting neurological recovery and reducing disability. Umbilical cord-derived mesenchymal stem cells (UC-MSCs) have shown potential in preclinical studies for their neuroprotective and regenerative properties^7–10^. Numerous studies have explored the use of MSCs for treating various conditions. Recent studies have highlighted the potential of UC-MSCs for treating stroke-related neurological deficits. Song et al. (2013) reported significant improvements in the NIHSS score stroke patients one month after UC-MSC intervention^11^. Jiang et al. (2013) confirmed the safety of UC-MSCs in four patients, noting muscle function improvements without major adverse events^12^. Shen et al. (2015) and Cai et al. (2015) further demonstrated the efficacy of UC-MSCs in enhancing neurological recovery and functional independence after intravenous administration^13^^,^^14^. Meta-analyses by Chen et al. (2016) and Xue et al. (2018) supported these findings, reporting significant improvements across the National Institutes of Health Stroke Scale (NIHSS), Barthel Index (BI), Fugl-Meyer Assessment (FMA), and Functional Independence Measure (FIM) scores with no severe adverse events^15^^,^^16^. More recent studies, including those by Kumar et al. (2021) and Huang et al. (2024), confirmed the safety of UC-MSC therapy, although the functional benefits varied, suggesting the need for further research to optimize administration methods and dosages^17^^,^^18^. These studies provide a foundation for further investigations into the potential benefits of UC-MSCs for stroke recovery. This study aimed to evaluate the safety and efficacy of UC-MSC infusion in improving neurological outcomes in patients with neurological sequelae at the subacute and chronic phases after ischemic stroke.

## Materials and methods

### Study design

This study has two designs: (1) a randomized study was applied to compare the safety and efficacy of intravenous (IV) versus intrathecal (IT) administration of cells, and (2) a matched control study was used to compare the safety and efficacy of cell therapy with those of controls not receiving cell therapy.

### Patients

Patients were selected on the basis of the following inclusion criteria:

- Age between 40 and 75 years.
- The patient was diagnosed with neurological sequelae after ischemic stroke, and met the following conditions:
  - Stable conditions without the need for vasopressors, mechanical ventilation, or oxygen therapy.
  - There were no cases of active infection, organ failure, or severe systemic disease.
- The time from stroke onset to UC-MSC transplantation was > 7 days and ≤ 24 months.
- NIHSS score ≥ 5.
- Informed consent to participate in the study was provided.

The exclusion criteria were as follows:

- Hemorrhagic or cardioembolic stroke.
- Active infections; heart, lung, liver, or kidney failure; respiratory failure; anemia; or coagulation disorders.
- Cancer.
- Pregnancy.
- Tracheostomy, coma, or persistent vegetative state.

### Study setting

The study was conducted at Vinmec Times City International Hospital from 2021-2024.

## Intervention

### Preparation and characterization of UC-MSCs

UC-MSCs were sourced from a high-quality master biobank, which was isolated and screened from 30 umbilical cords of full-term, healthy newborns free from infectious diseases, including HIV, cytomegalovirus, Epstein-Barr virus, hepatitis A virus, hepatitis B virus, hepatitis C virus, syphilis, and chlamydia^19^. The isolated UC-MSCs were cultured in serum-free and animal component-free conditions, as described previously^20^. One UC-MSC line was selected from this master bank for patient infusion, expanded to passage 5 (P5), and cryopreserved in CryoStore CS10 reagent (Stem Cell Technology, Canada) in the vapor phase of liquid nitrogen at -196°C in a Brooks system (Brooks Life Science, USA) until infusion. Cell viability was assessed via trypan blue, and sterility testing was conducted to exclude bacterial, fungal, and mycoplasma contamination. Additionally, MSC surface markers, including CD73, CD90, and CD105, and hematopoietic markers (CD45, CD34, CD19, CD11b, and HLA-DR) were analyzed on a Navios flow cytometer (Beckman Coulter, USA) via a Human MSC Analysis Kit (BD Biosciences, USA) according to the manufacturer’s instructions. The differentiation potential of UC-MSCs was assessed by their ability to undergo osteogenesis, adiposegenesis, and chondrogenesis in StemPro™ differentiation media (Gibco, USA). Differentiated cells were stained with Alizarin Red S, Oil Red O, and Alcian blue to detect osteocytes, adiposecytes, and chondrocytes, respectively.

The cells were thawed prior to infusion, washed to remove the CryoStore CS10 reagent, and suspended at a concentration of 1.5 × 10^6^ cells per kilogram of body weight in 10 ml Ringer’s lactate for IT administration or 50 ml for IV administration. Cell viability, endotoxin levels, and bacterial, fungal, and mycoplasma contamination were tested. The release criteria for UC-MSC products were as follows: the number of UC-MSCs was 1.5 × 10^6^ cells per kilogram of body weight, with cell viability exceeding 70%. Endotoxin levels were ≤ 0.2 EU/kg body weight for IT administration or ≤ 5 EU/kg body weight for IV administration. Bacterial, fungal, and mycoplasma contamination was absent. The expression of positive markers (CD73, CD90, and CD105) was ≥ 95%, while the expression of hematopoietic markers (CD45, CD34, CD11b, CD19, and HLA-DR) was ≤ 2%.

### Administration of UC-MSCs

For the IT infusion, UC-MSCs were thawed and infused over 30 minutes into the spinal canal at the L4-L5 intervertebral space via a lumbar puncture technique. Each patient received a dose of 1.5 × 10^6^ cells/kg of body weight, and the procedure was repeated three months later.

For IV infusion, UC-MSCs were thawed in the same manner and administered through a peripheral vein at the same dosage, timing, and infusion duration.

### Rehabilitation therapy

The traditional rehabilitation therapy for all groups consisted of 30 sessions tailored to each patient’s needs, with a focus on physical, occupational, and speech therapy. These sessions were designed to improve motor function, cognitive ability, and overall quality of life. Each session lasted 60 minutes.

### Follow-up and monitoring

All patients were followed for a period of 12 months postintervention, with follow-up visits scheduled at baseline, 3 months, 6 months, and 12 months.

### Outcome measures

Safety was assessed by monitoring adverse events (AEs) and serious adverse events (SAEs) throughout the study^21^ according to CTCAE version 4.03. Efficacy was evaluated via the NIHSS, FIM, Modified Ashworth Scale (MAS), Fine Motor Skills (FMS), SF-36 Health Survey, and brain MRI.

The NIHSS was used to assess neurological deficits in stroke patients, providing a comprehensive evaluation of various neurological functions, including consciousness, eye movements, visual fields, facial palsy, motor strength, limb ataxia, sensory loss, language, speech, and attention. Higher scores indicate greater neurological impairment. Scores are categorized as: 0 (no stroke symptoms), 1-4 (minor stroke), 5-15 (moderate stroke), 16-20 (moderate to severe stroke), and 21-42 (severe stroke). Our inclusion criterion of NIHSS ≥5 ensured enrollment of patients with moderate to severe stroke deficits^22^.

The Functional Independence Measure (FIM) was used to quantify disability and the level of assistance required for activities of daily living. The scale comprises 18 items scored on a 7‑level ordinal scale (1 = total assistance; 7 = complete independence) across two domains: Motor (13 items: self‑care, sphincter control, transfers, locomotion) and Cognitive (5 items: communication, social cognition). Higher scores denote greater functional independence. Domain ranges are 13–91 (Motor) and 5–35 (Cognitive), yielding a total FIM of 18–126. For clinical context, totals >108 suggest potential for home independence, whereas totals <40 indicate severe dependence requiring total assistance^23^.

The MAS (range: 0-4 points) was used to measure muscle spasticity in patients. This scale evaluates the resistance encountered during passive soft-tissue stretching, which reflects muscle tone abnormalities. Higher scores indicate greater muscle spasticity that can significantly impair mobility and comfort. Scores are interpreted as: 0 (no increase in muscle tone), 1 (slight increase in muscle tone with catch-and-release or minimal resistance), 1+ (slight increase in muscle tone with catch followed by minimal resistance throughout <50% of range of motion), 2 (more marked increase in muscle tone but affected parts easily moved), 3 (considerable increase in muscle tone making passive movement difficult), and 4 (affected parts rigid in flexion or extension). Higher baseline MAS scores reflect greater spasticity severity requiring targeted intervention^24^.

The FMS assessment focused on evaluating hand and finger dexterity, including fine gripping, pinching, manipulation of small objects, and hand-eye coordination. Each hand is assessed using a 26-item scale scored from 1 to 4, with an additional 3 sensory items scored from 0 to 2. The total score per hand ranges from 26 to 110, where higher scores indicate better fine motor function. The baseline score represents the initial level of fine motor skills, and subsequent assessments show the degree of improvement over time based on this scale, allowing clear understanding of changes in dexterity and coordination^25^.

The SF-36 (range: 0–100 points) was used to evaluate health-related quality of life across eight domains: physical functioning, role limitations due to physical problems, bodily pain, general health perceptions, vitality, social functioning, role limitations due to emotional problems, and mental health. Higher scores indicate better perceived health status and well-being. Scores are interpreted as: 0–20 (very poor quality of life), 21–40 (poor), 41–60 (fair), 61–80 (good), and 81–100 (excellent). Lower baseline SF-36 scores reflect greater impact of stroke sequelae on daily functioning and overall quality of life^26^.

Brain MRI was used to assess improvements in patients with neurological sequelae after ischemic stroke via the arterial spin labeling (ASL) technique. ASL was chosen for its ability to provide detailed information about cerebral blood flow (CBF) without requiring contrast agents, enabling noninvasive evaluation of cerebral perfusion. The ASL technique employs magnetic labeling of arterial blood to measure CBF directly, allowing for a comparison of cerebral perfusion before and after treatment, particularly in ischemic regions. Increased ASL values after treatment were interpreted as signs of improved cerebral perfusion, potentially correlating with clinical recovery. MRI scans were performed at 6- and 12-months posttreatment with UC-MSCs to evaluate changes in CBF^27^.

### Sample size

In previous research, the average FIM score for IV delivery of UC-MSCs was reported to be 21.9 ± 10.0 ^14^. We expect that the average FIM score for the IT delivery of UC-MSCs will be 32.0. With the Type I error probability (α) set at 0.05 and a power of 80%, the sample size was calculated via the following formula, accounting for a 10% dropout rate. Therefore, a total of 32 patients were recruited and randomized into two intervention groups: 16 patients received UC-MSCs via IT infusion plus rehabilitation, and 16 patients received UC-MSCs via IV infusion plus rehabilitation. Additionally, 16 matched control patients who received rehabilitation therapy alone, were matched one-to-one with patients in the IT group on the basis of sex, age (±5 years), and baseline NIHSS level.

### Randomization and allocation concealment

Patients were randomly assigned to one of two intervention groups—IV or IT UC-MSC infusion using a computer-generated randomization sequence. A block randomization method, with variable block sizes of 4 and 6, was applied in a 1:1 ratio. The randomization sequence was generated by an independent statistician and concealed using sequentially numbered, opaque, sealed envelopes. These envelopes were opened only after informed consent had been obtained and baseline assessments were completed, ensuring allocation concealment. Patient enrollment was carried out by study investigators who were blinded to the allocation sequence. A designated study coordinator, not involved in outcome assessments, was responsible for assigning patients to either the IV or IT group.

The control group was not randomized but was instead formed by matching each patient in the IT group to a control participant in a 1:1 ratio. Matching criteria included sex, age (±5 years), and baseline NIHSS level, ensuring that the control group was comparable to the IT group in key demographic and clinical characteristics.

### Data analysis

Baseline characteristics were summarized via descriptive statistics. Group differences in categorical variables, such as sex and stages of stroke recovery, were assessed via the chi-square test. For continuous variables, including age and clinical scores (NIHSS, FIM, MAS, FMS, and SF-36), one-way ANOVA was used if the data followed a normal distribution, or the Kruskal-Wallis test was applied if the data did not meet normality assumptions. The mixed-effects models were applied to the longitudinal data on the NIHSS, FIM, MAS, FMS, and SF-36 scores to analyze treatment efficacy over time. These models included fixed effects for the treatment groups (IV, IT, and control) and random intercepts for individual participants to account for within-subject correlations. Analyses were conducted via STATA software and statistical significance was set at p < 0.05.

Composite outcome at patient level: To characterize patient‑level heterogeneity at 12 months and provide a global summary of response, each outcome (NIHSS, MAS, FIM, FMS, SF‑36) was standardized to a baseline‑anchored z‑score using the cohort’s baseline mean and SD, and the within‑patient change from baseline to 12 months (Δz_12m) was computed. The 12‑month patient‑level composite was defined as the unweighted mean of aligned Δz_12m across available domains, yielding an effect‑size metric in SD units; a distribution‑based threshold of Δz_12m ≥0.5 SD was used to depict “responders” in rank‑ordered bar plots, consistent with minimal important change conventions.

## Results

Thirty-two patients received UC-MSCs and were randomly assigned to either the IV group (n = 16) or the IT group (n = 16). An additional 16 patients served as the control group, and were matched one-to-one with patients in the IT group based on gender, age (±5 years), and baseline NIHSS score. Patients in both the IT and IV groups received two stem cell infusions, spaced three months apart. At the time of the second infusion, one patient in the IT group did not receive treatment. Nonetheless, all patients—including those in the control group—were monitored and assessed at 3-, 6-, and 12-months post treatment (Figure S1).

### Patient characteristics

The characteristics of the participants at baseline in the three groups in terms of sex, age, time from onset to study participation, stages of stroke recovery, NIHSS score, FIM score, MAS score, FMS score, and SF-36 score are presented in Table 1.

**Table 1.** Clinical characteristics of patients at baseline.

Characteristics	IV (*n* = 16)	IT (*n* = 16)	Control group (*n* = 16)	*p*
**Gender, *N* (%)**				
***Male***	12 (75)	9 (56.3)	9 (56.3)	0.449
***Female***	4 (25)	7 (43.8)	7 (43.8)	
**Age, year, Mean (*SD*)**	56.5 (9.7)	62.4 (9.1)	62.2 (9.4)	0.1461
**Time from onset to study participation, month, Mean (*SD*)**	9.2 (6.1)	9.6 (5.9)	8.0 (7.5)	0.7795
**Stages of stroke recovery, *N* (%)**				
***Subacute phase (7 days to 6 months)***	7 (43.8)	6 (37.5)	7 (43.8)	0.918
***Chronic phase (>6 months)***	9 (56.2)	10 (62.5)	9 (56.2)	
**NIHSS score, Mean (*SD*)**	9.8 (4.9)	8.7 (4.5)	8.8 (3.4)	0.7085
**FIM score, Mean (*SD*)**	67.4 (21.8)	71.5 (15.7)	78.8 (17.2)	0.2199
**MAS score, Mean (*SD*)**	1.1 (0.9)	0.9 (0.5)	0.6 (0.3)	0.075
**FMS right-hand, Mean (*SD*)**	74.8 (34.5)	64.1 (37.7)	75.3 (35.5)	0.6161
**FMS left-hand, Mean (*SD*)**	82.8 (34.9)	79.1 (37.1)	88.3 (30.5)	0.7483
**SF-36, Mean (*SD*)**	34.1 (14.5)	34.4 (14.0)	41.9 (14.1)	0.2237

* *Note*: IV, Intravenous; IT= Intrathecal; NIHSS= National Institutes of Health Stroke Scale; FIM= Functional Independence Measure; MAS= Modified Ashworth Scale; FMS= Fine Motor Skills; SF-36= Short-form 36 items.

In the IT group, lesions most commonly affected the left middle cerebral artery (50.0%), followed by the right side (31.3%) and both sides (6.3%). In the IV group, the distribution differed slightly, with bilateral middle cerebral artery involvement in 37.5% of patients, followed by the left side (31.3%) and the right side (18.8%). In the control group, the left middle cerebral artery was the most commonly affected area (43.8%), followed by the right side (31.3%) and both sides (25.0%) (Table S1).

### Cell viability and quality assessment

UC-MSCs exhibited a normal adherent and spindle-shaped morphology (Figure S2A). The potential of UC-MSCs to differentiate into osteogenic, chondrogenic, and adipogenic lineages was analyzed to demonstrate their multipotency (Figure S2B). Furthermore, UC-MSCs highly expressed CD73, CD90, and CD105, but lacked of hematopoietic markers (CD45, CD34, CD19, CD11b, and HLA-DR) (Figure S2C).

During the first infusion, the IV group (n = 16) had a viability rate of 87.7 ± 4.8%, whereas the IT group (n = 16) had a slightly greater viability rate of 89.7 ± 2.7%. After the second infusion, the cell viability was 91.1 ± 3.3% in the IV group (n = 16) and 91.0 ± 3.4% in the IT group (n = 15). All the samples were free of bacteria, fungi, and mycoplasma contamination and had acceptable endotoxin levels.

### Safety evaluation

A total of four severe adverse events (SAEs) were observed in three patients. In the IV group, two SAEs related to COVID-19 infection were reported. In the IT group, two SAEs occurred: one case of gastrointestinal bleeding caused by a gastric ulcer and another requiring bioprosthetic mitral valve replacement. None of these SAEs were related to UC-MSC therapy.

In the IV group, four adverse events (AEs) were documented, including three cases of headache with visual analog scale (VAS) scores ranging from 1 to 4 points, and one case of redness and slight swelling at the infusion site.

In the IT group, a total of 17 AEs were reported. These patients comprised 11 patients with mild pain at the procedure site (VAS score of 1–3) and 4 patients with mild to moderate headache (VAS score of 1–4). Other recorded AEs included 1 case of mild sacral pain (VAS score of 2) following the first infusion and 1 case of hypertension (160/72 mmHg), accompanied by mild body aches and exacerbated pain with positional changes after the second infusion.

## Efficiency evaluation

### Improvements in the NIHSS score

The mixed-effects model indicated that both the IV and IT groups showed improvements in NIHSS scores over time, with varying degrees of improvement compared with the control group at different time points. At 3 months, the IV group had a reduction of 1.7 points (95% CI: [-4.2, 0.8]; p = 0.179) compared with the control group, whereas the IT group had a reduction of 1.5 points (95% CI: [-3.9, 0.9]; p = 0.215), although neither reached statistical significance. By 6 months, the IV group demonstrated a statistically significant reduction of 2.5 points (95% CI: [-5.0, -0.04]; p = 0.046) compared with the control group, whereas the IT group exhibited a reduction of 2.3 points (95% CI: [-4.6, 0.1]; p = 0.063), approaching significance. By 12 months, both groups showed significant improvements compared with the control group, with the IV group demonstrating a reduction of 4.5 points (95% CI: [-7.0, -2.0]; p < 0.001) and the IT group showing a reduction of 3.6 points (95% CI: [-6.0, -1.3]; p = 0.003).

Although both the IV and IT groups demonstrated significant improvements in NIHSS scores over time compared with the control group, direct comparisons between the two treatment methods revealed no statistically significant differences at any analyzed time point (p > 0.05) (Table 2).

**Table 2.** Comparison of the NIHSS score over time between groups via a mixed-effects model.

Model Parameters	IV vs control			IT vs control			IT vs IV		
	Estimate ± SE	95% CI	p	Estimate ± SE	95% CI	p	Estimate ± SE	95% CI	p
**Constant**	8.8 ± 1.2	[6.3,11.2]	0.541	8.8 ± 1.2	[6.4, 11.1]	0.971	9.8 ± 1.1	[7.6, 12.0]	0.476
**Baseline treatment (*IV vs Control or IT vs Control or IT vs IV*)**	1.1 ± 1.7	[-2.3, 4.5]		-0.1 ± 1.7	[-3.4, 3.3]		-1.1 ± 1.6	[-4.2, 2.0]	
**Time point # Treatment group**									
**3 months # UC-MSC group**	-1.7 ± 1.3	[-4.2, 0.8]	0.179	-1.5 ± 1.2	[-3.9, 0.9]	0.215	0.2 ± 0.8	[-1.3, 1.7]	0.807
**6 months # UC-MSC group**	-2.5 ± 1.3	[-5.0, -0.04]	0.046	-2.3 ± 1.2	[-4.6, 0.1]	0.063	0.3 ± 0.8	[-1.3, 1.8]	0.745
**12 months # UC-MSC group**	-4.5 ± 1.3	[-7.0, -2.0]	0.000	-3.6 ± 1.2	[-6.0, -1.3]	0.003	0.9 ± 0.8	[-0.6, 2.4]	0.254

* *Note*: UC-MSCs, Umbilical cord-derived mesenchymal stem cells; IV, Intravenous; IT, Intrathecal. ‘Constant’ represents the baseline NIHSS score. ‘Baseline treatment’ indicates the estimated difference in baseline NIHSS scores between groups (IV vs Control, IT vs Control, IT vs IV). ‘Time point × treatment group’ represents the estimated change in NIHSS scores at 3, 6, and 12 months for each treatment group. Negative estimates indicate improvement in NIHSS scores.

### Improvements in the FIM scores

Compared with the control group, both the IV and IT groups showed significant improvements in overall FIM scores, as well as motor and cognitive function FIM scores over time. For overall FIM scores, at 3 months, the IV group increased by 5.0 points (95% CI: [-3.1, 13.1]; p = 0.227), and the IT group increased by 2.2 points (95% CI: [-6.8, 11.3]; p = 0.627). By 6 months, the IV group showed a significant increase of 9.1 points (95% CI: [1.0, 17.2]; p = 0.028), whereas the IT group increased by 4.5 points (95% CI: [-4.6, 13.6]; p = 0.331). At 12 months, both groups achieved substantial increases, with the IV group increasing by 17.9 points (95% CI: [9.8, 26.0]; p < 0.001) and the IT group increasing by 17.1 points (95% CI: [8.0, 26.1]; p < 0.001). When the IV and IT groups were compared directly, there were no significant differences at any time point (p > 0.05) (Table 3).

**Table 3.** Comparison of FIM scores over time between groups via a mixed-effects model.

Model Parameters	IV vs control			IT vs control			IT vs IV		
	Estimate ± SE	95% CI	p	Estimate ± SE	95% CI	p	Estimate ± SE	95% CI	p
**Constant**	78.8 ± 5.2	[68.6, 89.1]	0.125	78.8 ± 4.6	[69.9, 87.7]	0.257	67.4 ± 4.8	[58.1, 76.8]	0.547
**Baseline treatment (*IV vs Control or IT vs Control or IT vs IV*)**	-11.4 ± 7.4	[-25.9, 3.1]		-7.3 ± 6.5	[-19.9, 5.3]		4.1 ± 6.8	[-9.2, 17.3]	
**Time point # Treatment group**									
**3 months # UC-MSC group**	5.0 ± 4.1	[-3.1, 13.1]	0.227	2.2 ± 4.6	[-6.8, 11.3]	0.627	-2.8 ± 3.4	[-9.3, 3.8]	0.413
**6 months # UC-MSC group**	9.1 ± 4.1	[1.0, 17.2]	0.028	4.5 ± 4.6	[-4.6, 13.6]	0.331	-4.6 ± 3.4	[-11.1, 2.0]	0.174
**12 months # UC-MSC group**	17.9 ± 4.1	[9.8, 26.0]	0.000	17.1 ± 4.6	[8.0, 26.1]	0.000	-0.8 ± 3.4	[-7.4, 5.8]	0.809

* *Note*: UC-MSCs, Umbilical cord-derived mesenchymal stem cells; IV, Intravenous; IT, Intrathecal. ‘Constant’ represents the baseline FIM score. ‘Baseline treatment’ indicates the estimated difference in baseline FIM scores between groups (IV vs Control, IT vs Control, IT vs IV). ‘Time point × treatment group’ represents the estimated change in FIM scores at 3, 6, and 12 months for each treatment group.

For motor function FIM scores, at 3 months, the scores of the IV group increased by 3.4 points (95% CI: [-2.9, 9.7]; p = 0.295), and those of the IT group increased by 1.4 points (95% CI: [-6.0, 8.8]; p = 0.715). At 6 months, the IV group showed a significant increase of 6.6 points (95% CI: [0.3, 12.9]; p = 0.04), whereas the IT group increased by 2.9 points (95% CI: [-4.5, 10.3]; p = 0.436). By 12 months, both groups showed significant increases, with the IV group increasing by 12.1 points (95% CI: [5.8, 18.4]; p < 0.001) and the IT group increasing by 11.9 points (95% CI: [4.5, 19.3]; p = 0.002). However, there were no significant differences between the IV and IT groups (p > 0.05) (Table 4).

**Table 4.** Comparison of changes in motor function FIM scores over time between groups via a mixed-effects model.

Model Parameters	IV vs control			IT vs control			IT vs IV		
	Estimate ± SE	95% CI	p	Estimate ± SE	95% CI	p	Estimate ± SE	95% CI	p
**Constant**	56.6 ± 4.5	[47.9; 65.4]	0.209	56.6 ± 3.5	[49.9; 63.4]	0.187	48.7 ± 4.2	[40.5; 56.9]	0.801
**Baseline treatment (*IV vs Control or IT vs Control or IT vs IV*)**	-7.9 ± 6.3	[-20.3; 4.4]		-6.4 ± 4.9	[-16.6; 3.8]		1.5 ± 6.0	[-10.2; 13.2]	
**Time point # Treatment group**									
**3 months # UC-MSC group**	3.4 ± 3.2	[-2.9; 9.7]	0.295	1.4 ± 3.8	[-6.0; 8.8]	0.715	-2.0 ± 3.0	[-7.8; 3.8]	0.497
**6 months # UC-MSC group**	6.6 ± 3.2	[0.3; 12.9]	0.04	2.9 ± 3.8	[-4.5; 10.3]	0.436	-3.7 ± 3.0	[-9.5; 2.1]	0.211
**12 months # UC-MSC group**	12.1 ± 3.2	[5.8; 18.4]	<0.001	11.9 ± 3.8	[4.5; 19.3]	0.002	-0.2 ± 3.0	[-6.0; 5.6]	0.949

* *Note*: UC-MSCs, Umbilical cord-derived mesenchymal stem cells; IV, Intravenous; IT, Intrathecal. ‘Constant’ represents the baseline motor function FIM score (average motor function FIM score across all groups). ‘Baseline treatment’ indicates the estimated difference in baseline motor function FIM scores between groups (IV vs Control, IT vs Control, IT vs IV). ‘Time point × treatment group’ represents the estimated change in motor function FIM scores at 3, 6, and 12 months for each treatment group.

For cognitive function FIM scores, at 3 months, the scores of the IV group increased by 1.6 points (95% CI: [-0.6, 3.8]; p = 0.146), and those of the IT group increased by 0.9 points (95% CI: [-1.4, 3.2]; p = 0.450). By 6 months, the score in the IV group had increased significantly by 2.4 points (95% CI: [0.2, 4.6]; p = 0.029), and that in the IT group had increased by 1.6 points (95% CI: [-0.7, 3.8]; p = 0.177). At 12 months, both groups had significant increases, with the IV group increasing by 5.8 points (95% CI: [3.6, 7.9]; p < 0.001) and the IT group increasing by 5.1 points (95% CI: [2.9, 7.4]; p < 0.001). No significant differences were observed between the two groups (p > 0.05) (Table 5).

**Table 5.** Comparison of changes in cognitive function FIM scores over time between groups via a mixed-effects model.

Model Parameters	IV vs control			IT vs control			IT vs IV		
	Estimate ± SE	95% CI	p	Estimate± SE	95% CI	p	Estimate ± SE	95% CI	p
**Constant**	22.2 ± 1.3	[19.6; 24.8]	0.066	22.2 ± 1.4	[19.4; 25.0]	0.655	18.8 ± 1.2	[16.4; 21.1]	0.131
**Baseline treatment (*IV vs Control or IT vs Control or IT vs IV*)**	-3.4 ± 1.9	[-7.1; 0.2]		-0.9 ± 2.0	[-4.7; 2.9]		2.6 ± 1.7	[-0.8; 5.9]	
**Time point # Treatment group**									
**3 months # UC-MSC group**	1.6 ± 1.1	[-0.6; 3.8]	0.146	0.9 ± 1.2	[-1.4; 3.2]	0.45	-0.8 ± 0.8	[-2.3; 0.8]	0.343
**6 months # UC-MSC group**	2.4 ± 1.1	[0.2; 4.6]	0.029	1.6 ± 1.2	[-0.7; 3.8]	0.177	-0.9 ± 0.8	[-2.4; 0.6]	0.269
**12 months # UC-MSC group**	5.8 ± 1.1	[3.6; 7.9]	<0.001	5.1 ± 1.2	[2.9; 7.4]	<0.001	-0.6 ± 0.8	[-2.2; 0.9]	0.429

* *Note*: UC-MSCs, Umbilical cord-derived mesenchymal stem cells; IV, Intravenous; IT, Intrathecal. ‘Constant’ represents the baseline cognitive function FIM score. ‘Baseline treatment’ indicates the estimated difference in baseline cognitive function FIM scores between groups (IV vs Control, IT vs Control, IT vs IV). ‘Time point × treatment group’ represents the estimated change in cognitive function FIM scores at 3, 6, and 12 months for each treatment group.

### Changes in the MAS scores

The mixed-effects model results indicate that both the IV and IT groups showed improvements in Modified Ashworth Scale (MAS) scores over time. At 3 months, the IV group demonstrated a decrease of 0.2 points (95% CI: [-0.5, 0.0]; p = 0.104), while the IT group showed a similar decrease of 0.2 points (95% CI: [-0.4, 0.0]; p = 0.117), although neither reached statistical significance.

By 6 months, the IV group achieved a significant reduction of 0.4 points (95% CI: [-0.6, -0.1]; p = 0.012) compared with the control group, whereas the IT group exhibited a nonsignificant reduction of 0.1 points (95% CI: [-0.5, 0.2]; p = 0.316). At 12 months, both groups demonstrated significant reductions, with the IV group showing a decrease of 0.7 points (95% CI: [-0.9, -0.4]; p < 0.001) and the IT group showing a decrease of 0.3 points (95% CI: [-0.5, 0.0]; p = 0.028) (Table 6).

**Table 6.** Comparison of MAS score changes over time among the UC-MSC infusion groups via either the IV or IT route and the control via the mixed-effects model.

Model Parameters	IV vs control			IT vs control		
	Estimate ± SE	95% CI	p	Estimate ± SE	95% CI	p
**Constant**	0.6 ± 0.2	[0.2, 0.9]	0.015	0.6 ± 0.1	[0.3, 0.8]	0.07
**Baseline treatment (IV or IT vs Control)**	0.6 ± 0.2	[0.1, 1.0]		0.3 ± 0.2	[-0.0, 0.6]	
**Time point # Treatment group**						
**3 months # UC-MSC group**	-0.2 ± 0.1	[-0.5, 0.0]	0.104	-0.2 ± 0.1	[-0.4, 0.0]	0.117
**6 months # UC-MSC group**	-0.4 ± 0.1	[-0.6, -0.1]	0.012	-0.1 ± 0.1	[-0.4, 0.2]	0.316
**12 months # UC-MSC group**	-0.7 ± 0.1	[-0.9, -0.4]	<0.001	-0.3 ± 0.1	[-0.5, -0.0]	0.028

* *Note*: UC-MSCs, Umbilical cord-derived mesenchymal stem cells; MAS, Modified Ashworth Scale; IV, Intravenous; IT, Intrathecal. ‘Constant’ represents the baseline MAS score. ‘Baseline treatment’ indicates the estimated difference in baseline MAS scores between groups (IV vs Control, IT vs Control). ‘Time point × treatment group’ represents the estimated change in MAS scores at 3, 6, and 12 months for each treatment group.

### Improvements in FMS scores

#### FMS right-hand scores

Compared to the control group, both the IV and IT groups showed consistent improvements in FMS right-hand scores over time. At 3 months, the score of IV group increased by 6.3 points (95% CI: [-2.0, 14.5]; p = 0.139), whereas that of the IT group increased by 3.4 points (95% CI: [-5.7, 12.4]; p = 0.465). By 6 months, the IV group achieved a statistically significant increase of 13.3 points (95% CI: [5.0, 21.5]; p = 0.002), whereas the IT group showed a nonsignificant increase of 8.4 points (95% CI: [-0.6, 17.5]; p = 0.068). At 12 months, both groups demonstrated significant improvements, with the IV group increasing by 17.1 points (95% CI: [8.8, 25.3]; p < 0.001) and the IT group increasing by 16.8 points (95% CI: [7.8, 25.9]; p < 0.001). No significant differences were found between the IV and IT groups in FMS right-hand scores at any time point (p > 0.05) (Table S2A).

#### FMS left-hand scores

Compared to the control group, both the IV and IT groups showed improvements in FMS left-hand scores over time. At 3 months, the IV group increased by 2.6 points (95% CI: [-7.8, 12.9]; p = 0.628), whereas the IT group increased by 0.5 points (95% CI: [-9.9, 10.8]; p = 0.925). By 6 months, the IV group increased by 5.0 points (95% CI: [-5.4, 15.4]; p = 0.344), and the IT group increased by 2.0 points (95% CI: [-8.3, 12.3]; p = 0.706). At 12 months, the IV group approached statistical significance with an increase of 10.1 points (95% CI: [-0.2, 20.5]; p = 0.055), whereas the IT group demonstrated a significant increase of 11.4 points (95% CI: [1.0, 21.8]; p = 0.032). No significant differences in FMS left-hand scores were observed between the IV and IT groups at any time point (p > 0.05) (Table S2B).

In patients with bilateral MCA involvement, mixed‐effects analysis of left‐hand FMS scores (Table S2C) revealed no significant Time point × Treatment group interactions at 3, 6, or 12 months for IV versus control or IT versus control (all p > 0.05).

### SF-36 health survey (SF36)

The mixed-effects model indicated significant improvements in SF-36 scores over time in both the IV and IT groups compared with the control group. At 3 months, the IV group demonstrated a substantial increase of 22.5 points (95% CI: [11.1, 33.9]; p < 0.001), whereas the IT group showed an improvement of 17.8 points (95% CI: [6.3, 29.3]; p = 0.002).

By 6 months, the upward trend continued for both groups, with the IV group improving by 26.3 points (95% CI: [14.9, 37.7]; p < 0.001) and the IT group increasing by 17.2 points (95% CI: [5.7, 28.7]; p = 0.003). At 12 months, the IV group achieved a further increase of 32.1 points (95% CI: [20.7, 43.4]; p < 0.001), whereas the IT group continued to improve, with a rise of 21.0 points (95% CI: [9.6, 32.5]; p < 0.001) (Table 7).

**Table 7.** Comparison of SF-36 general health score changes over time among the UC-MSC infusion groups (IV, IT) and the control group (mixed-effects model).

Model Parameters	IV vs control			IT vs control		
	Estimate ± SE	95% CI	p	Estimate ± SE	95% CI	p
**Constant**	41.9 ± 3.9	[34.3, 49.5]	0.153	41.9 ± 3.6	[34.8, 48.9]	0.14
**Treatment group (*IV vs Control or IT vs Control*)**	-7.8 ± 5.5	[-18.5, 2.9]		-7.5 ± 5.1	[-17.5, 2.5]	
**Time point # Treatment group**						
**3 months # UC-MSC group**	22.5 ± 5.8	[11.1, 33.9]	<0.001	17.8 ± 5.9	[6.3, 29.3]	0.002
**6 months # UC-MSC group**	26.3 ± 5.8	[14.9, 37.7]	<0.001	17.2 ± 5.9	[5.7, 28.7]	0.003
**12 months # UC-MSC group**	32.1 ± 5.8	[20.7, 43.4]	<0.001	21.0 ± 5.9	[9.6, 32.5]	<0.001

* *Note*: UC-MSCs, Umbilical cord-derived mesenchymal stem cells; IV, Intravenous; IT, Intrathecal; SF-36= Short-form 36 items. ‘Constant’ represents the baseline SF-36 score. ‘Baseline treatment’ indicates the estimated difference in baseline SF-36 scores between groups (IV vs Control, IT vs Control). ‘Time point × treatment group’ represents the estimated change in SF-36 scores at 3, 6, and 12 months for each treatment group.

### Patient-level composite and responders at 12 months

At 12 months, patient‑level baseline‑standardized changes showed consistent positive shifts in both IV and IT arms versus controls across all domains, with many participants exceeding the 0.5 SD reference line indicated in the plots. For FIM, most IV and IT patients were above 0 and a notable subset surpassed 0.5–1.0 SD, whereas controls were more dispersed with several declines. For MAS and NIHSS (direction‑reversed for display), IV patients clustered above 0 with multiple exceeding 0.5 SD, IT exhibited smaller but steady positive shifts, and controls had values around 0 with some negatives. For SF‑36, both intervention arms shifted upward with many surpassing 0.5 SD, while controls mainly trended below or near 0. For FMS (right and left hands), IV and IT distributions moved to the positive side with several patients exceeding 0.5 SD, whereas controls showed scattered bars near or below 0, highlighting domain‑specific gains in fine motor dexterity (Figure S3- Figure S8).

### MRI of the brain

Brain MRI was used to evaluate improvements in cerebral perfusion on the basis of baseline ASL values. At 6 months, 3/16 (18.8%) patients in the IV group showed improved perfusion, whereas 2/16 (12.5%) patients in both the IT and control groups improved (p = 0.846). By 12 months, the IV group had 4/16 (25%) patients who improved, compared with 18.8% in the control group and 2/16 (12.5%) in the IT group (p = 0.663).

## Discussion

To our knowledge, this is the first randomized study to compare IV and IT administration of cells for ischemic stroke in both the subacute and chronic phases. This study provides additional evidence supporting the safety of UC-MSC therapy for stroke. No SAEs related to UC-MSC therapy were observed during the study period. All AEs in both the IV and IT groups were minor and either resolved spontaneously or responded to medication. However, AEs occurred more frequently in the IT group than in the IV group, mainly due to pain at the lumbar puncture site. Our findings are consistent with those of other studies. In 2024, Tang Y et al. published a meta-analysis of six randomized clinical trials investigating the use of bone marrow mononuclear cells (BMMNCs) for ischemic stroke. The analysis included 177 patients who received BMMNC infusions and 166 patients who did not. The authors reported no significant differences between the two groups in terms of AEs, including fever, infection, seizures, and cardiac disorders^28^. In the same year, Shen Z et al. conducted a meta-analysis of 30 studies on MSC therapies for ischemic stroke, comprising 624 patients in the intervention group and 593 patients in the control group. The authors found no significant differences in AEs between the two groups. Furthermore, the mortality rate was lower in the cell therapy group than in the control group^29^.

Our study demonstrated that UC-MSC therapy, administered via either the IV or IT route, led to significant improvements in patients with neurological sequelae from ischemic stroke during the subacute and chronic stages. These improvements were observed in neurological function, motor ability, muscle tone, and quality of life. By 12 months, both the IV and IT groups showed remarkable improvements in the NIHSS, FIM, FMS, MAS, and SF-36 scores compared with those of the control group, with statistically significant differences. These findings align with the results of other studies. In 2023, Fauzi AA et al. conducted a meta-analysis of 21 studies involving 836 patients, including 406 who received stem cell therapy. Most of these patients are in the subacute or chronic phase of stroke. The authors reported improvements in the NIHSS, mRS, and BI scores following stem cell therapy^30^. Another meta-analysis conducted by Shen Z et al. in 2024 highlighted the benefits of cell therapy for stroke during the subacute and chronic phases. Researchers have reported that MSCs significantly improved the NIHSS score when administered 2 weeks to 3 months after ischemic stroke onset. Notably, these improvements in NIHSS scores continued when MSCs were administered more than 3 months poststroke^29^.

In contrast to the subacute and chronic phases of stroke, several studies have shown that cell therapy during the acute phase is less effective. Shen Z et al. reported no significant improvements in the mRS, NIHSS, or BI scores when cell therapy was administered within 2 weeks of stroke onset^29^. Similarly, in a randomized study involving 104 patients who received allogeneic stem cells and 102 who received a placebo, Houkin K et al. reported that intravenous administration of allogeneic mesenchymal stem cells within 18–36 hours of stroke onset did not improve short-term outcomes^31^.

Our study demonstrated that improvements in neurological sequelae occurred gradually following cell infusion. By three months postintervention, significant changes in NIHSS scores compared with those of the control group were not observed in either the IV or the IT group. By six months, the improvement in the NIHSS score was significant in the IV group and reached a significant level in the IT group. Notably, by 12 months, improvements in the NIHSS score were significant in both groups. A similar trend was observed for FIM scores, MAS scores, FMS hand scores, and SF-36 scores.

Although we hypothesized that IT cell therapy would yield better outcomes than IV administration, the study results did not support this assumption. Improvements across all parameters were not significantly different between the two groups at any time point after the intervention. The efficacy of cell therapy based on the route of administration has also been explored in other studies. In 2023, Fauzi AA et al. published a meta-analysis comparing the efficacy of three different administration routes for stem cell therapy in ischemic stroke. In this analysis, stem cells were administered intravenously in 304 patients (78.8%), via intracerebral injection in 64 patients (16.5%), and intra-arterially in 38 patients (9.35%)^30^. The results indicated that intracerebral administration led to better outcomes than the other routes did, although it was associated with a higher incidence of adverse events due to its invasiveness. Our study confirmed a higher rate of adverse events in the IT cell therapy group; however, the clinical outcomes were comparable between the two groups.

MSCs can support recovery of the central venous system after ischemic stroke through various mechanisms. Key therapeutic mechanisms include immunomodulation and the secretion of growth factors such as brain-derived neurotrophic factor (BDNF), glial cell-derived neurotrophic factor (GDNF), and nerve growth factor (NGF) to improve neurological recovery and prevent neuron death. Additionally, MSCs promote angiogenesis and enhance neural circuit reconstruction^32^^,^^33^. Recent studies have indicated that MSCs also transfer mitochondria to restore mitochondrial function in injured cells^34^^,^^35^.

Our study evaluated cerebral perfusion via MRI and revealed no significant difference between the cell therapy groups and the control group, suggesting that cell therapy does not increase brain perfusion. Although MSCs are known to enhance angiogenesis via increased expression of angiogenic factors such as vascular endothelial growth factor (VEGF), platelet-derived growth factor (PDGF), angiopoietin-1 (Ang1), and Tie-2^32^, our data did not reveal a superior proangiogenic effect of infused UC-MSCs, which were administered either intrathecally or intravenously.

While significant progress has been made in implementing cell therapy for stroke treatment, the biological actions of MSCs depending on the timing of administration remain to be elucidated. Many animal studies have focused on the acute phase of stroke, which is characterized by various cellular and molecular tissue damage reactions due to disruption of the blood supply to the brain^36^. During this phase, cell therapy aims to reduce neuroinflammation, oxidative stress, and cell death^37^. The subsequent repair and remodeling of the damaged brain increase neuroplasticity and restore blood-brain barrier integrity^38^. In a study by Lee et al., neuroimaging of stroke patients after intravenous infusion of bone marrow-derived MSCs revealed improved neural connectivity and reduced degeneration of the corticospinal tract, which controls motor functions^39^. Further research is essential to gain a deeper understanding of the impact of MSCs on the functional recovery of patients with subacute and chronic stroke. This knowledge could pave the way for more innovative and effective clinical applications in treating the later phases of stroke, where treatment options are currently limited.

Although our study provides evidence supporting the efficacy of cell therapy for patients with neurological sequelae following ischemic stroke, it is limited by certain limitations, notably the small sample size, which may limit the broader applicability of the findings.

Our trial showed 12‑month gains of 17–18 points on total FIM and 21–32 points on SF‑36, but the economic value of these improvements is unknown. Future studies should prospectively collect direct and indirect costs across the full care pathway (manufacture, storage, infusion, rehabilitation use, readmissions, long‑term care) and perform cost‑effectiveness and cost‑utility analyses. These should report incremental cost‑effectiveness ratios as cost per FIM point gained and cost per QALY, deriving utilities from SF‑36 via validated mapping, and quantify uncertainty using non‑parametric bootstrapping with cost‑effectiveness acceptability curves.

## Conclusion

Both IV and IT administration of UC-MSCs improved neurological sequelae in the subacute and chronic phases of ischemic stroke. However, the IV administration route is associated with fewer adverse events than the IT route is. A randomized trial with a larger sample size is needed to draw more accurate conclusions.

## Ethics approval and consent to participate

The study adhered to the ethical principles outlined in the Declaration of Helsinki and complied with ICH/GCP guidelines and local regulations. Informed consent was obtained from all participants. The clinical trial was approved by the Ethics Committee of Vinmec International Hospital [approval number 48/2020/QĐ-VMEC] and the Vietnam Ministry of Health [approval number 3322/QĐ-BYT]. This study was registered at ClinicalTrials.gov [ID: NCT05292625]. All participants were not required to pay fees related to cell culture, cell infusion, hospital stays, laboratory tests, MRI scans, rehabilitation therapy or follow-up visits.

## Supplementary Material

szaf063_Supplementary_Data

## Data Availability

The data that support the findings of this study are available on request from the corresponding author.
