# Supplementary material for: Intrathecal versus intravenous umbilical cord mesenchymal stem cells for ischemic stroke sequelae
Source: Stem Cells Transl Med. 2025 Nov 24;14(12):szaf063. doi: 10.1093/stcltm/szaf063 (PMC12641229; doi:10.1093/stcltm/szaf063)
Supplement: szaf063_Supplementary_Data [file szaf063_supplementary_data.zip › Figure S1.docx]

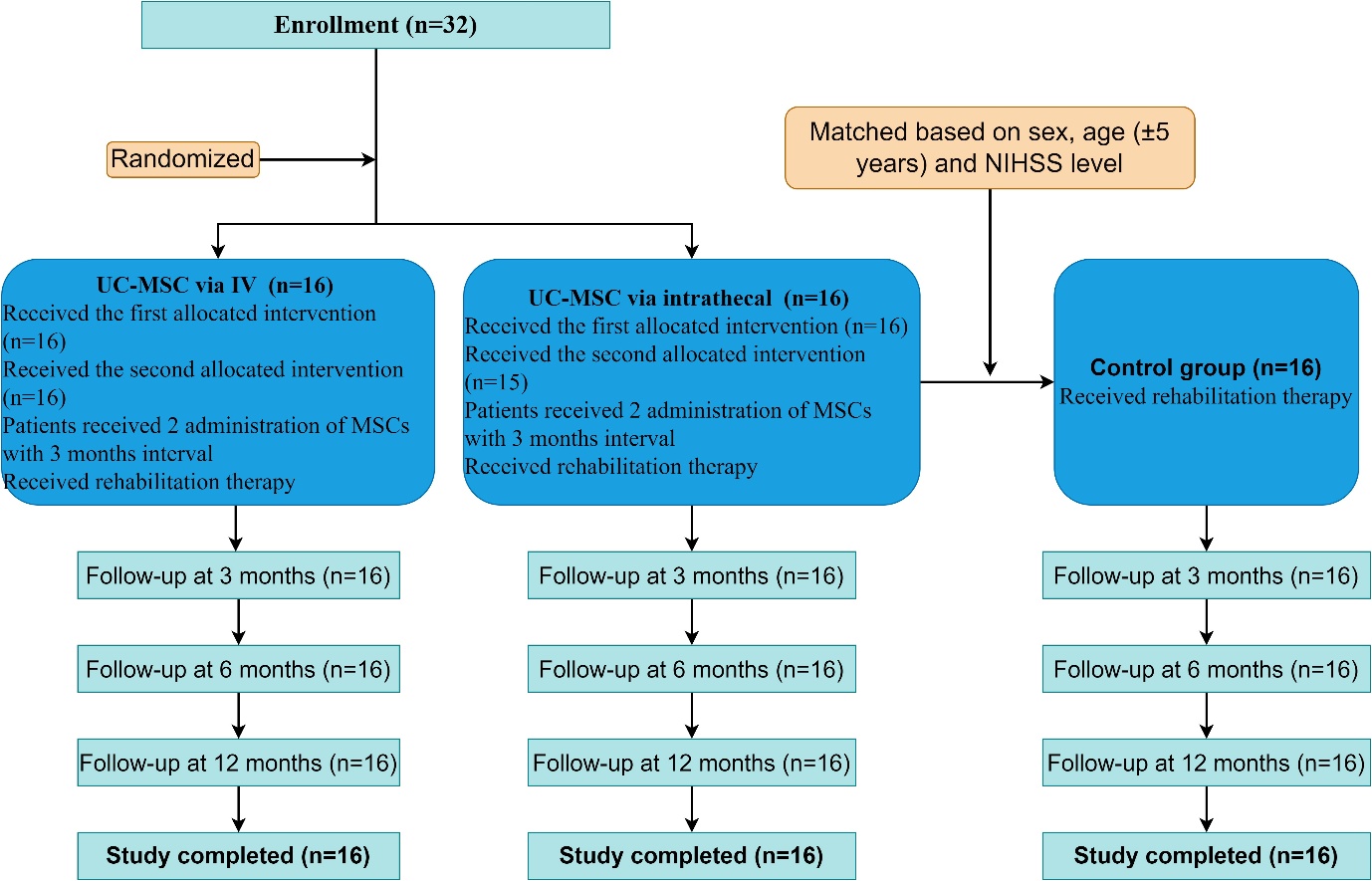


***** One patient in the intrathecal group refused the second stem cell infusion

**Figure S1. Consort diagram of the study**

*Figure legend*. This flow diagram illustrates the enrollment, randomization, and follow-up processes of a phase II clinical trial assessing the safety and efficacy of allogeneic umbilical cord-derived mesenchymal stem cell (UC-MSC) infusions delivered via intravenous (IV) and intrathecal (IT) routes for treating neurological sequelae after ischemic stroke. A total of 32 patients were enrolled and randomized into two treatment arms: UC-MSC infusion via IV administration (n=16) or IT administration (n=16). Additionally, a matched control group (n=16), stratified by sex, age (±5 years), and NIHSS level, received rehabilitation therapy alone. Participants in the intervention arms received two UC-MSC infusions (1.5 × 10⁶ cells/kg) at baseline and at three months, combined with rehabilitation therapy. One patient in the IT group declined the second infusion but completed the initial treatment. Follow-up assessments were conducted at 3, 6, and 12 months across all groups. By the conclusion of the study, all 48 participants completed follow-up assessments.
