# Supplementary material for: Intrathecal versus intravenous umbilical cord mesenchymal stem cells for ischemic stroke sequelae
Source: Stem Cells Transl Med. 2025 Nov 24;14(12):szaf063. doi: 10.1093/stcltm/szaf063 (PMC12641229; doi:10.1093/stcltm/szaf063)
Supplement: szaf063_Supplementary_Data [file szaf063_supplementary_data.zip › Figure S2.docx]

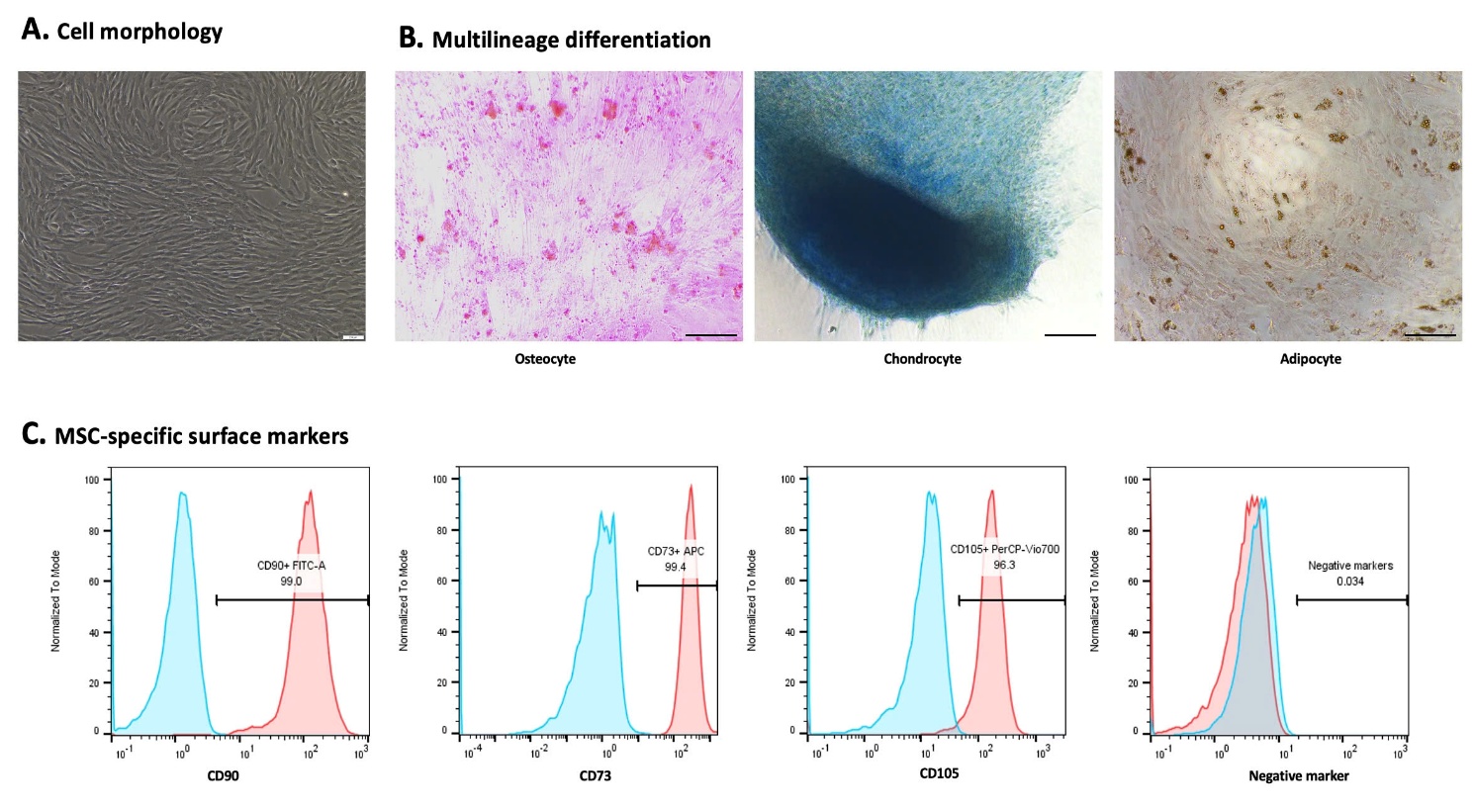


**Figure S2. Characterization of UC-MSCs**

*Figure legend*. A) The typical morphology of UC-MSCs was observed via phase-contrast microscopy. The scale bar represents 200 **μ**m. B) UC-MSCs were capable to differentiating into osteocytes, chondrocytes, and adipocytes, and the black scale bar represents 100 **μ**m. C) UC-MSCs expressed high levels of CD90, CD73, and CD 105 and low levels of negative markers (CD45, CD34, CD19, CD11b, and HLA-DR).
