# Supplementary material for: Intrathecal versus intravenous umbilical cord mesenchymal stem cells for ischemic stroke sequelae
Source: Stem Cells Transl Med. 2025 Nov 24;14(12):szaf063. doi: 10.1093/stcltm/szaf063 (PMC12641229; doi:10.1093/stcltm/szaf063)
Supplement: szaf063_Supplementary_Data [file szaf063_supplementary_data.zip › Figure S3.docx]

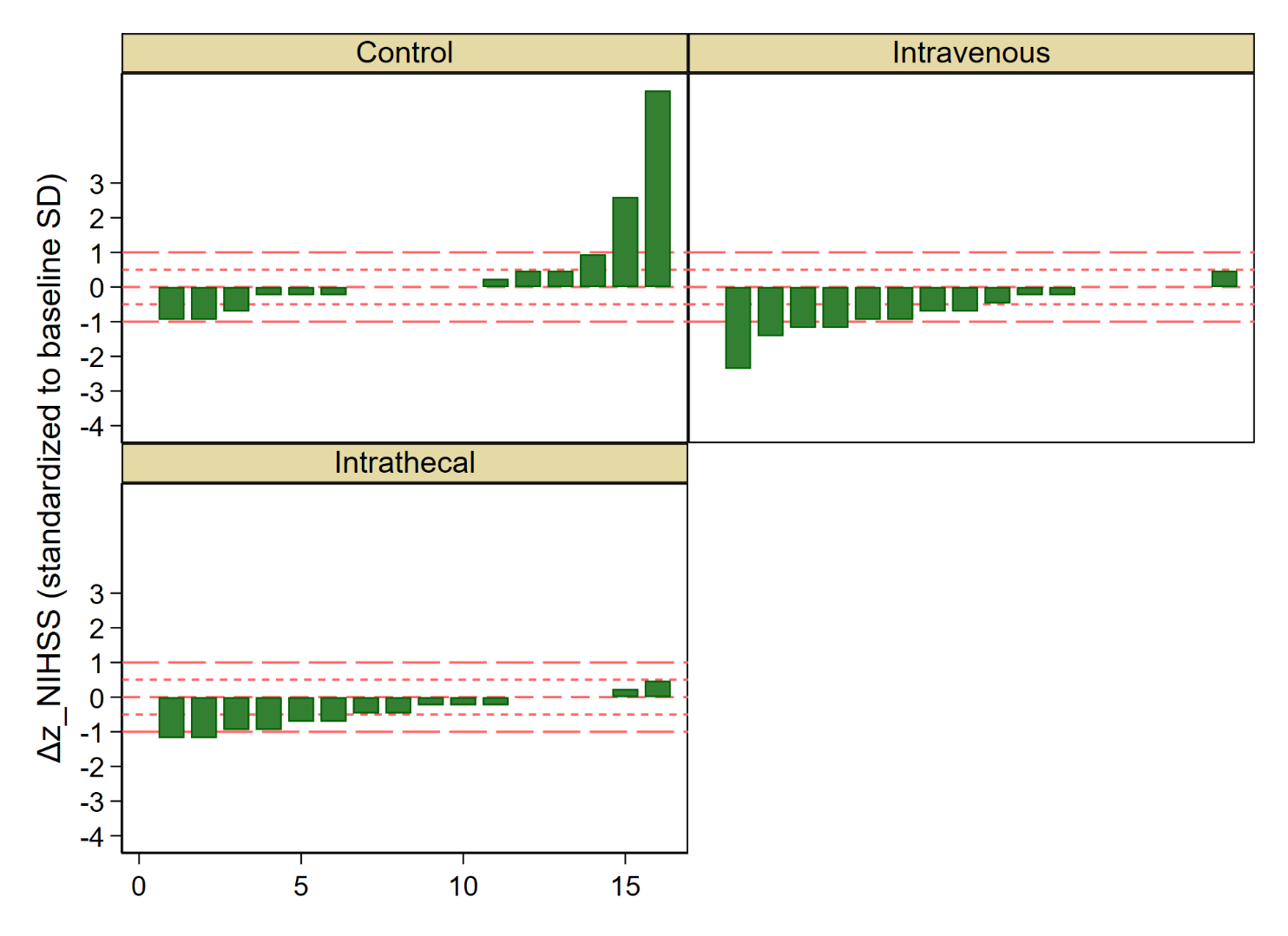


**Figure S3. Rank‑ordered patient change in NIHSS at 12 months (Δz standardized to baseline SD) between groups**

*Figure legend*: Each bar represents one participant ordered by Δz_NIHSS, downward bars indicate improvement defined as a lower neurological deficit versus baseline using the group’s baseline SD, dashed lines denote 0.5 SD and 1.0 SD reference thresholds, panels display Control, Intravenous infusion, and Intrathecal infusion at 12 months for visual comparison. Participants with no change from baseline (Δz = 0) are not visible on the plot.
