# Supplementary material for: Intrathecal versus intravenous umbilical cord mesenchymal stem cells for ischemic stroke sequelae
Source: Stem Cells Transl Med. 2025 Nov 24;14(12):szaf063. doi: 10.1093/stcltm/szaf063 (PMC12641229; doi:10.1093/stcltm/szaf063)
Supplement: szaf063_Supplementary_Data [file szaf063_supplementary_data.zip › Figure S4.docx]

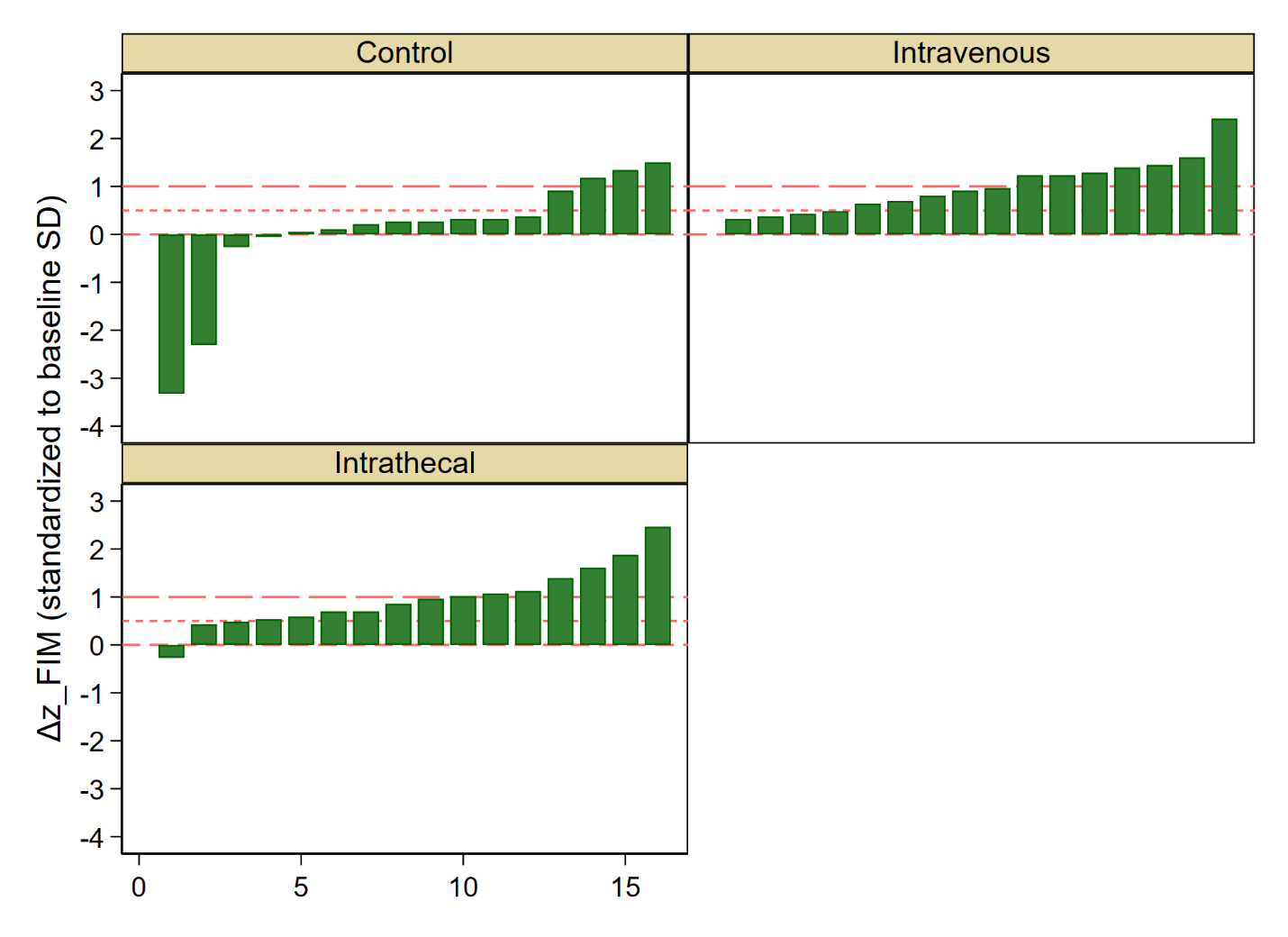


**Figure S4. Rank‑ordered patient change in FIM total at 12 months (Δz standardized to baseline SD) between groups**

*Figure legend*: Each bar represents one participant ranked by Δz_FIM total, upward bars indicate improvement defined as greater functional independence in activities of daily living, dashed lines denote 0.5 SD and 1.0 SD reference thresholds, panels display Control, Intravenous infusion, and Intrathecal infusion at 12 months.
