# Supplementary material for: Intrathecal versus intravenous umbilical cord mesenchymal stem cells for ischemic stroke sequelae
Source: Stem Cells Transl Med. 2025 Nov 24;14(12):szaf063. doi: 10.1093/stcltm/szaf063 (PMC12641229; doi:10.1093/stcltm/szaf063)
Supplement: szaf063_Supplementary_Data [file szaf063_supplementary_data.zip › Figure S6.docx]

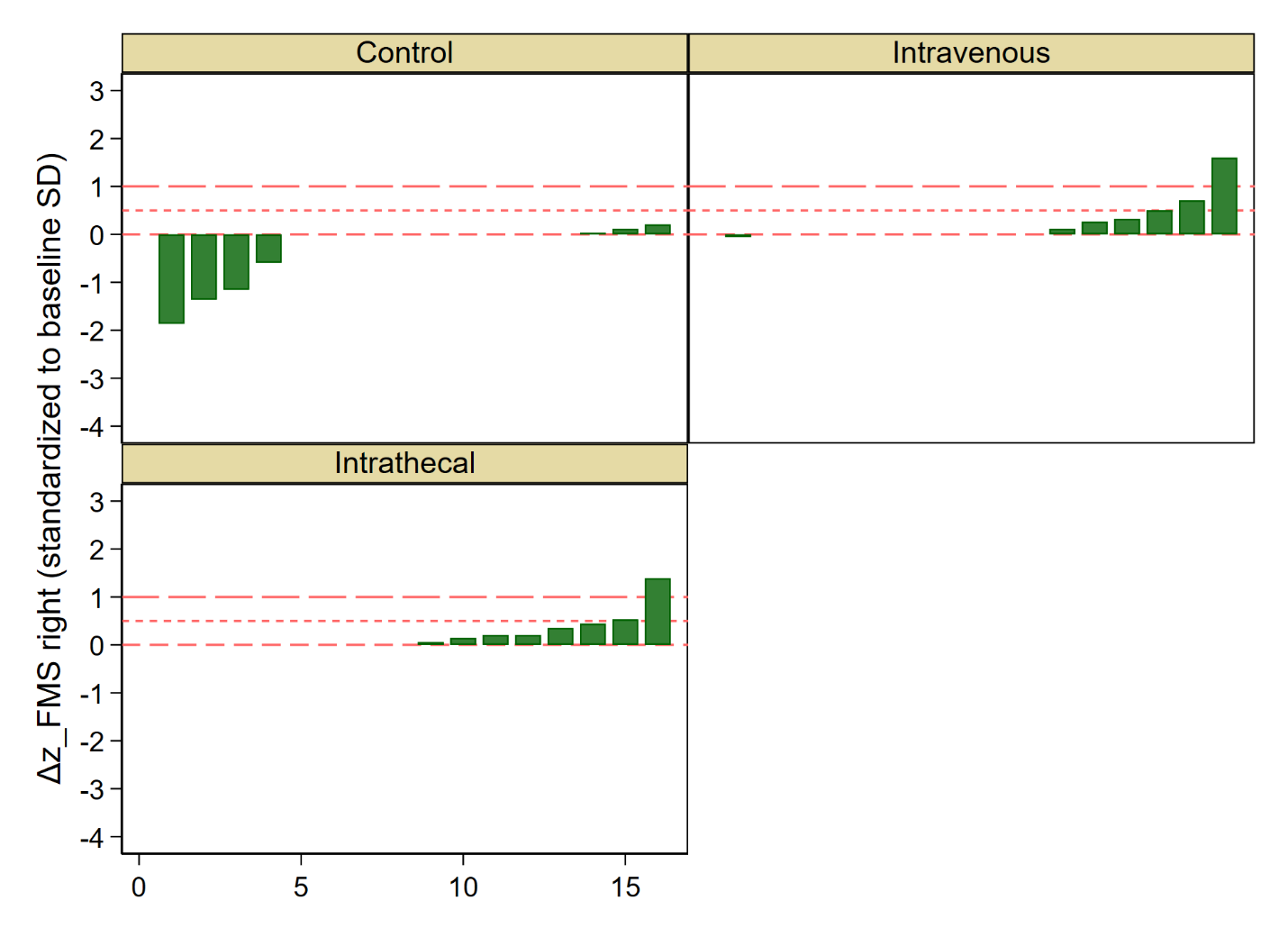


**Figure S6. Rank‑ordered patient change in FMS right at 12 months (Δz standardized to baseline SD) between groups**

*Figure legend*: Each bar is one participant ordered by Δz_FMS right, upward bars indicate improvement defined as better right‑hand dexterity versus baseline, dashed lines mark 0.5 SD and 1.0 SD, panels show Control, intravenous, and intrathecal at 12 months. Participants with no change from baseline (Δz = 0) are not visible on the plot.
