# Supplementary material for: Intrathecal versus intravenous umbilical cord mesenchymal stem cells for ischemic stroke sequelae
Source: Stem Cells Transl Med. 2025 Nov 24;14(12):szaf063. doi: 10.1093/stcltm/szaf063 (PMC12641229; doi:10.1093/stcltm/szaf063)
Supplement: szaf063_Supplementary_Data [file szaf063_supplementary_data.zip › Figure S7.docx]

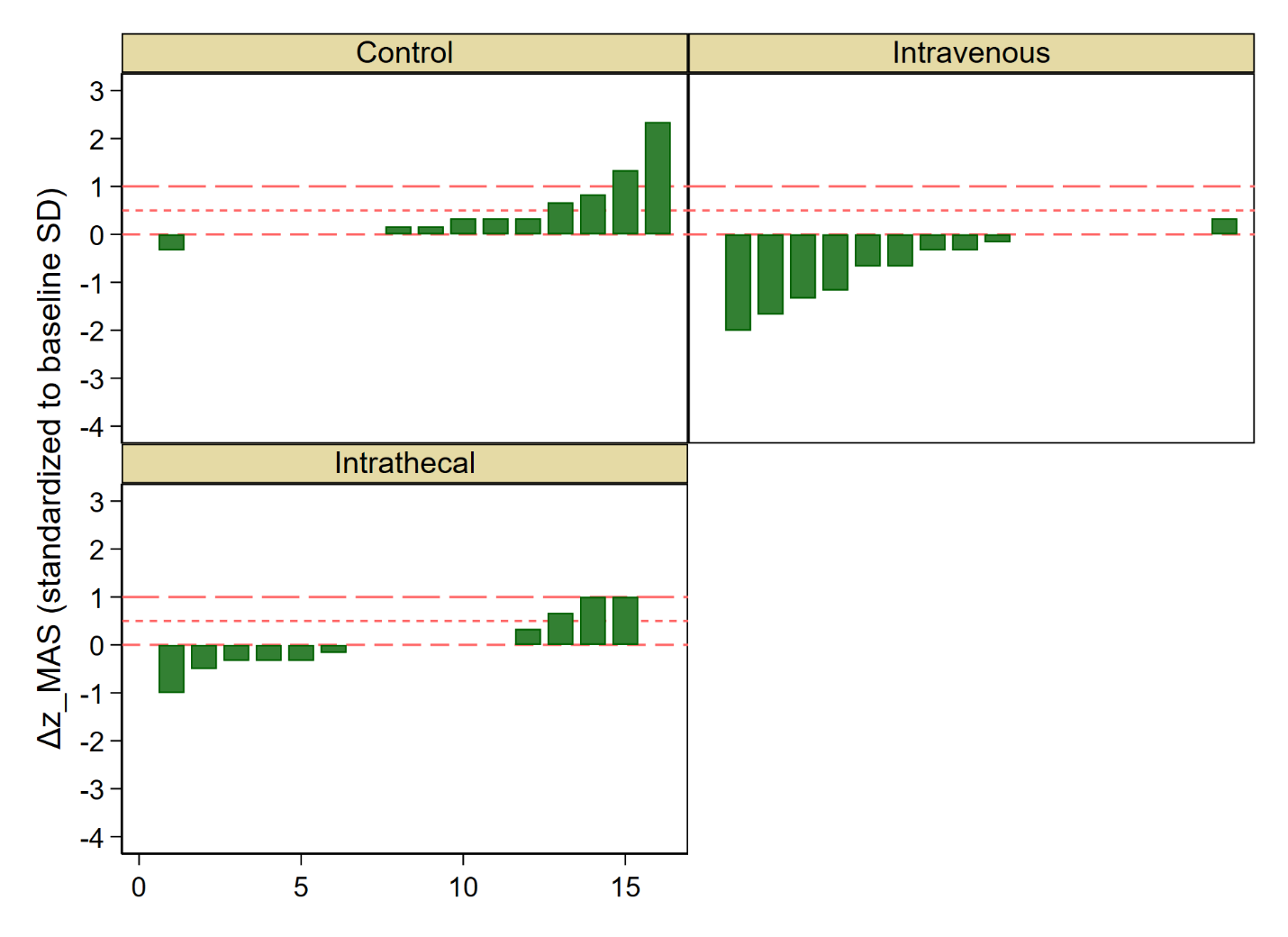


**Figure S7. Rank‑ordered patient change in MAS at 12 months (Δz standardized to baseline SD) between groups**

*Figure legend*: Each bar represents one participant ordered by Δz_MAS, downward bars indicate improvement defined as less spasticity versus baseline, dashed lines denote 0.5 SD and 1.0 SD reference thresholds, panels display Control, Intravenous, and Intrathecal at 12 months. Participants with no change from baseline (Δz = 0) are not visible on the plot.
