# Supplementary material for: Intrathecal versus intravenous umbilical cord mesenchymal stem cells for ischemic stroke sequelae
Source: Stem Cells Transl Med. 2025 Nov 24;14(12):szaf063. doi: 10.1093/stcltm/szaf063 (PMC12641229; doi:10.1093/stcltm/szaf063)
Supplement: szaf063_Supplementary_Data [file szaf063_supplementary_data.zip › Figure S8.docx]

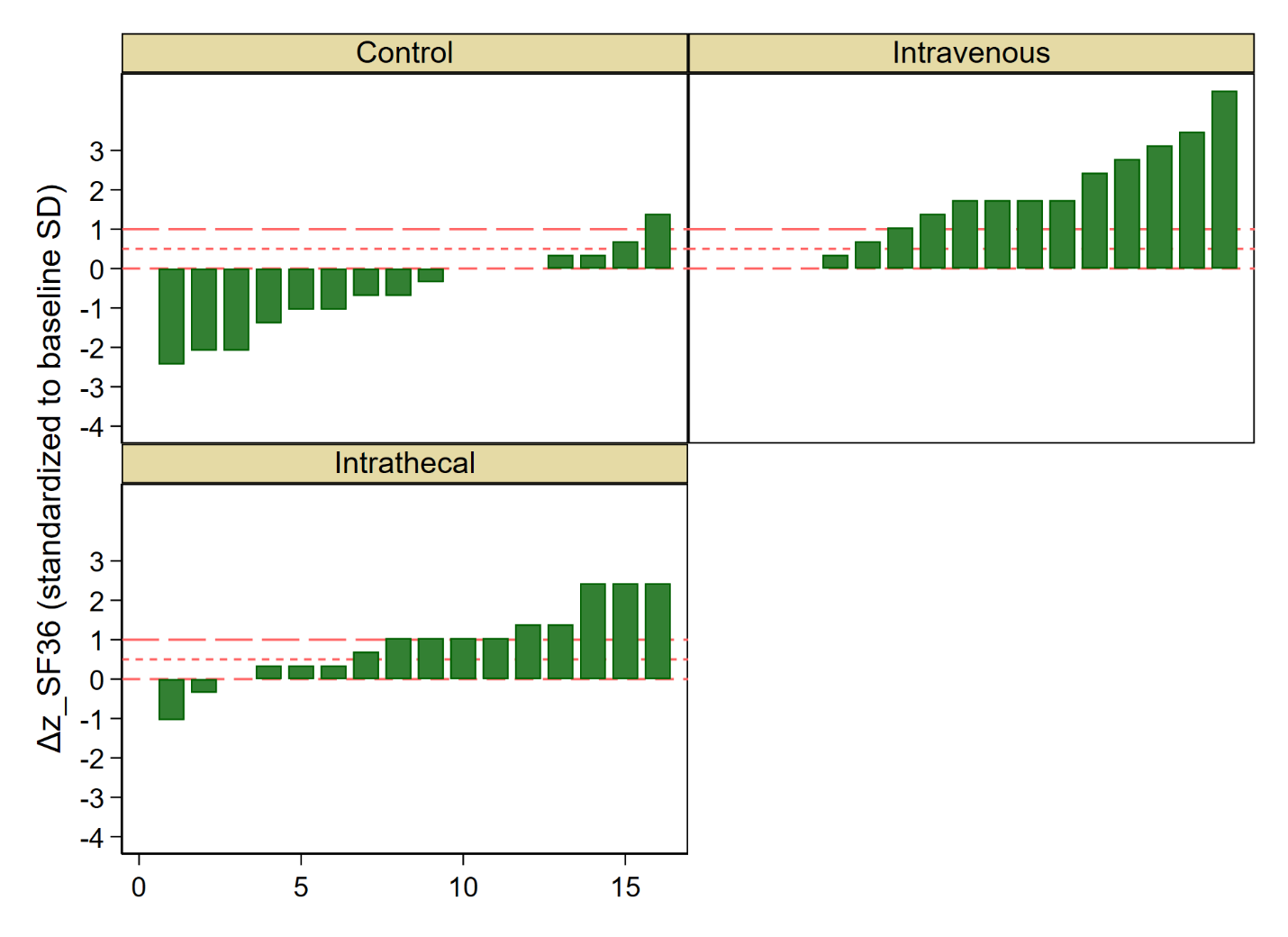


**Figure S8. Rank‑ordered patient‑level change in SF‑36 at 12 months (Δz standardized to baseline SD) between groups**

*Figure legend*: Each bar represents one participant ordered by Δz_SF36 upward bars indicate improvement defined as higher health‑related quality of life versus baseline using the group’s baseline SD dashed lines denote 0.5 SD and 1.0 SD reference levels panels display Control IV and IT side‑by‑side for visual comparison at 12 months. Participants with no change from baseline (Δz = 0) are not visible on the plot.
