# Supplementary material for: Intrathecal versus intravenous umbilical cord mesenchymal stem cells for ischemic stroke sequelae
Source: Stem Cells Transl Med. 2025 Nov 24;14(12):szaf063. doi: 10.1093/stcltm/szaf063 (PMC12641229; doi:10.1093/stcltm/szaf063)
Supplement: szaf063_Supplementary_Data [file szaf063_supplementary_data.zip › Supplementary Information.docx]

**Intrathecal versus Intravenous Umbilical Cord Mesenchymal Stem Cells for Ischemic Stroke Sequelae**

**Authors**: Liem Nguyen Thanh^1 3 Ϯ *^, Thuy Nguyen Thi Ngoc^2 Ϯ^ , Kien Nguyen Trung^1 Ϯ^, Lam Phung Nam^3^, Van Hoang Thanh^1^, Trang Phan Thi Kieu^1^, Minh Pham Van^2 4^, Anh Thi Phuong Nguyen ^3^, Doan Ngo Van^3^, Anh Nguyen Van^3^, Chi Nguyen Van^5^

**Affiliations:**

1. Vinmec Research Institute of Stem Cell and Gene Technology, College of Health Sciences, VinUniversity, Vinhomes Ocean Park, Gia Lam District, Hanoi, Vietnam
2. Ha Noi Medical University, 1 Ton That Tung, Dong Da District, Hanoi, Vietnam
3. Vinmec Times City International Hospital, Vinmec HealthCare System, 458 Minh Khai Street, Hai Ba Trung District, Hanoi, Vietnam
4. Ha Noi Rehabilitation Hospital, 35 Le Van Thiem, Thanh Xuan District, Hanoi, Vietnam
5. National Rehabilitation Hospital, 27 Le Loi, Sam Son, Thanh Hoa, Vietnam

^Ϯ^ These authors contributed equally and are considered co- first authors

***Correspondence**: Liem Nguyen Thanh, Vinmec Research Institute of Stem Cell and Gene Technology, College of Health Science, VinUniversity, Vinhomes Ocean Park, Gia Lam District, Hanoi 100000, Vietnam. Phone: 84(0)986.565.015; Email: liem.nt@vinuni.edu.vn


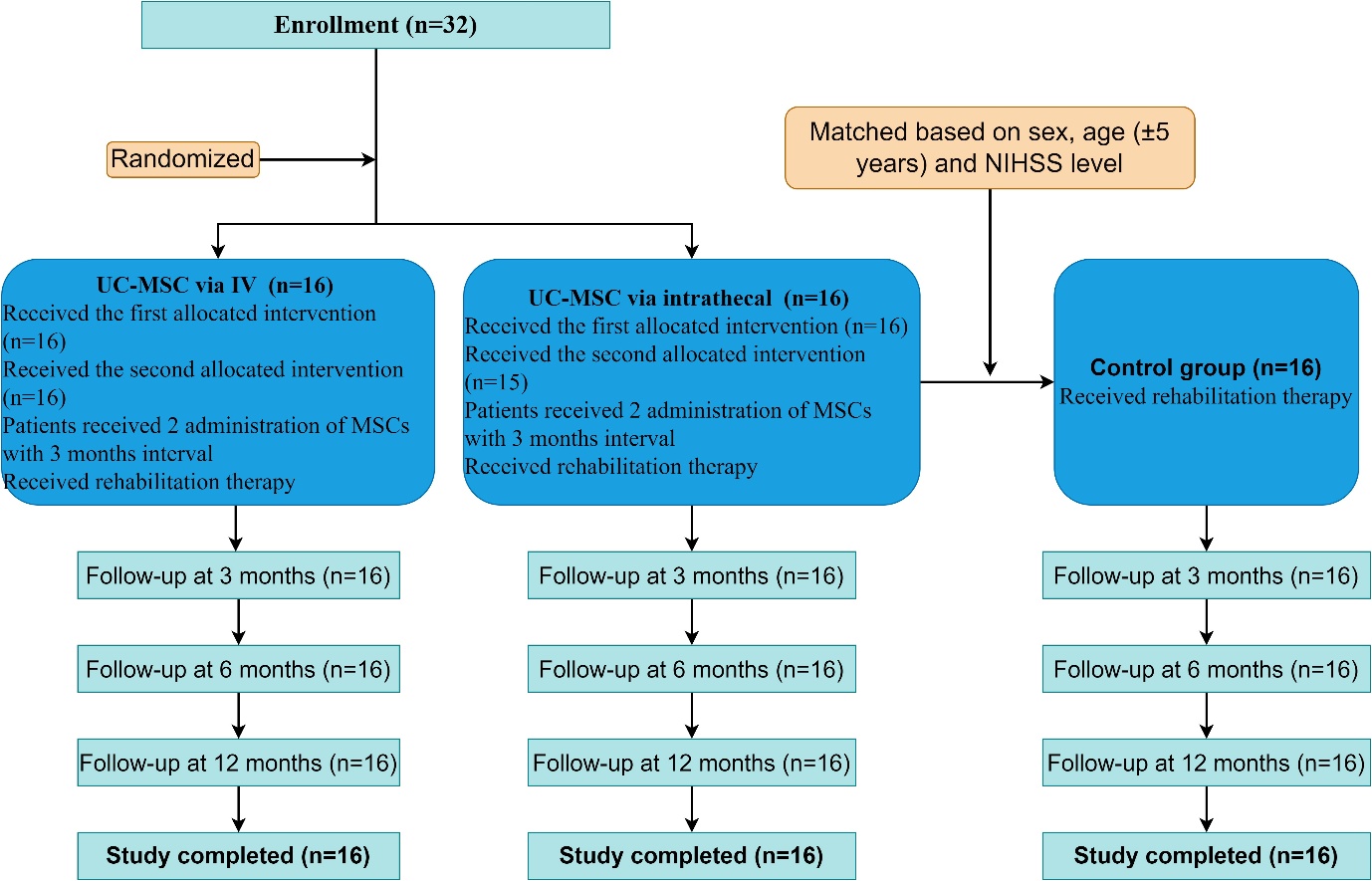


***** One patient in the intrathecal group refused the second stem cell infusion

**Figure S1. Consort diagram of the study**

*Figure legend*. This flow diagram illustrates the enrollment, randomization, and follow-up processes of a phase II clinical trial assessing the safety and efficacy of allogeneic umbilical cord-derived mesenchymal stem cell (UC-MSC) infusions delivered via intravenous (IV) and intrathecal (IT) routes for treating neurological sequelae after ischemic stroke. A total of 32 patients were enrolled and randomized into two treatment arms: UC-MSC infusion via IV administration (n=16) or IT administration (n=16). Additionally, a matched control group (n=16), stratified by sex, age (±5 years), and NIHSS level, received rehabilitation therapy alone. Participants in the intervention arms received two UC-MSC infusions (1.5 × 10⁶ cells/kg) at baseline and at three months, combined with rehabilitation therapy. One patient in the IT group declined the second infusion but completed the initial treatment. Follow-up assessments were conducted at 3, 6, and 12 months across all groups. By the conclusion of the study, all 48 participants completed follow-up assessments.

**Table S1. Lesion locations at baseline in each group**

| **Lesion location** | **IV**  **(n=16)** | **IT**  **(n=16)** | **Control**  **(n=16)** |
| --- | --- | --- | --- |
| Middle cerebral artery bilateral | 6 (37.5%) | 1 (6.3 %) | 4 (25.0%) |
| Middle cerebral artery - right | 3 (18.8%) | 5 (31.3 %) | 5 (31.3%) |
| Middle cerebral artery - left | 5 (31.3%) | 8 (50.0 %) | 7 (43.8%) |
| Bilateral anterior cerebral artery | 1 (6.3%) | 1 (6.3%) | 0 (0%) |
| Left + anterior cerebral artery | 1 (6.3%) | 1 (6.3%) | 0 (0%) |

**Note: IV = Intravenous; IT= Intrathecal; The data are presented as N (%)*


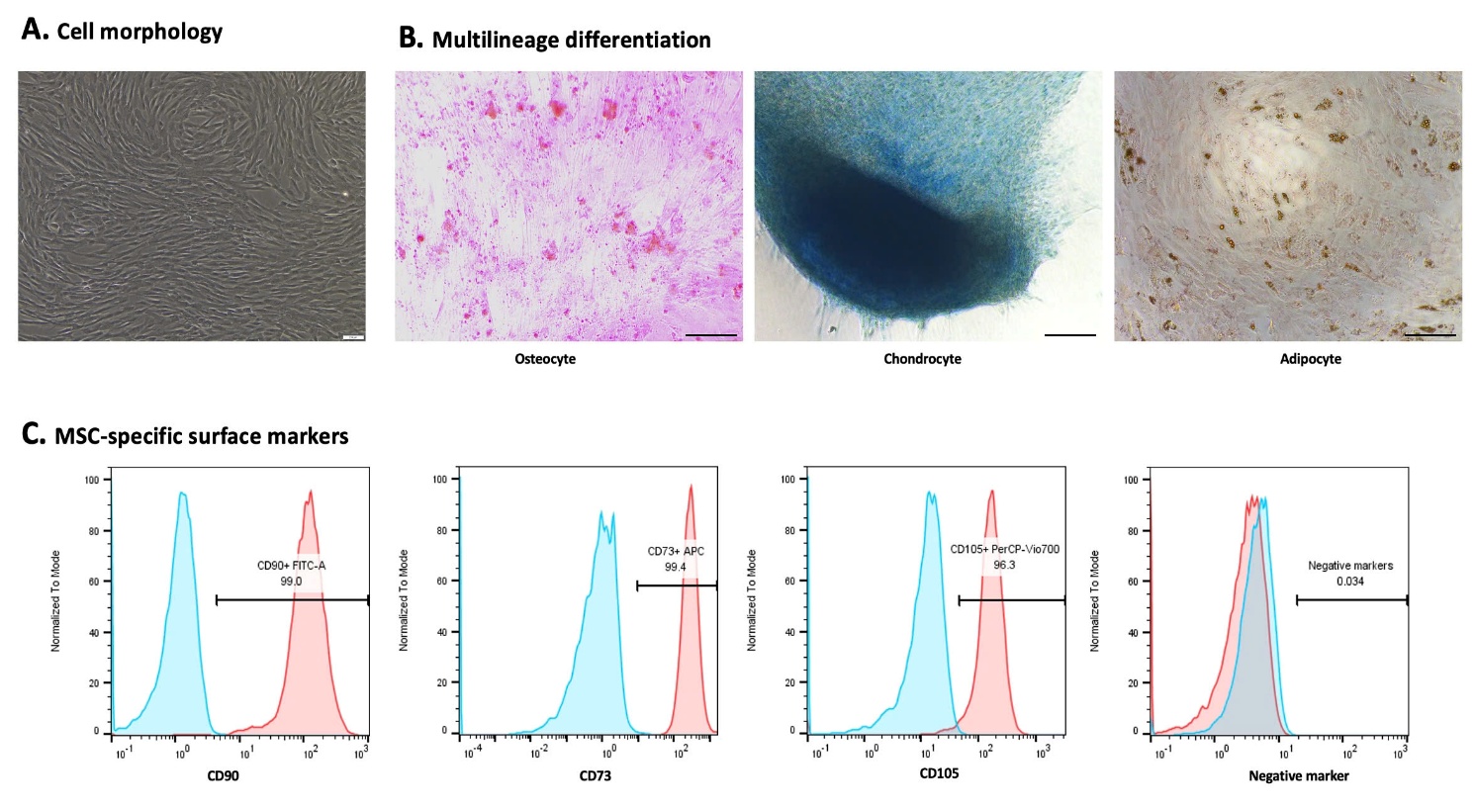


**Figure S2. Characterization of UC-MSCs**

*Figure legend*. A) The typical morphology of UC-MSCs was observed via phase-contrast microscopy. The scale bar represents 200 **μ**m. B) UC-MSCs were capable to differentiating into osteocytes, chondrocytes, and adipocytes, and the black scale bar represents 100 **μ**m. C) UC-MSCs expressed high levels of CD90, CD73, and CD 105 and low levels of negative markers (CD45, CD34, CD19, CD11b, and HLA-DR).

**Table S2A.** **Comparison of FMS right-hand scores over time between groups via a mixed-effects model**

| **Model Parameters** | **IV vs control** | | | **IT vs control** | | | **IT vs IV** | | |
| --- | --- | --- | --- | --- | --- | --- | --- | --- | --- |
|  | **Estimate ± SE** | **95% CI** | **p** | **Estimate ± SE** | **95% CI** | **p** | **Estimate ± SE** | **95% CI** | **p** |
| Constant | 75.3 ± 8.6 | [58.5, 92.0] | 0.967 | 75.3 ± 8.8 | [58.0, 92.5] | 0.372 | 74.8 ± 8.6 | [57.9, 91.6] | 0.382 |
| Baseline treatment  (*IV vs Control or IT vs Control or IT vs IV*) | -0.5 ± 12.1 | [-24.2, 23.2] |  | -11.1 ± 12.5 | [-35.5, 13.3] |  | -10.6 ± 12.2 | [-34.5, 13.2] |  |
| Time point # Treatment group |  |  |  |  |  |  |  |  |  |
| 3 months # UC-MSC group | 6.3 ± 4.2 | [-2.0, 14.5] | 0.139 | 3.4 ± 4.6 | [-5.7, 12.4] | 0.465 | -2.9 ± 4.2 | [-11.2, 5.4] | 0.497 |
| 6 months # UC-MSC group | 13.3 ± 4.2 | [5.0, 21.5] | 0.002 | 8.4 ± 4.6 | [-0.6, 17.5] | 0.068 | -4.8 ± 4.2 | [-13.1, 3.5] | 0.255 |
| 12 months # UC-MSC group | 17.1 ± 4.2 | [8.8, 25.3] | <0.001 | 16.8 ± 4.6 | [7.8, 25.9] | <0.001 | -0.3 ± 4.2 | [-8.5, 8.0] | 0.953 |

******* *Note: UC-MSCs = Umbilical cord-derived mesenchymal stem cells; FMS =* *Fine Motor Skills; IV = Intravenous; IT = Intrathecal. 'Constant' represents the baseline FMS right-hand score. 'Baseline treatment' indicates the estimated difference in baseline FMS right-hand scores between groups (IV vs Control, IT vs Control, IT vs IV). 'Time point × treatment group' represents the estimated change in FMS right-hand scores at 3, 6, and 12 months for each treatment group*

**Table S2B.** **Comparison of FMS changes in left-hand pain scores over time among the UC-MSC infusion groups via either the IV or IT route and the control group via the mixed-effects model**

| **Model Parameters** | **IV vs control** | | | **IT vs control** | | | **IT vs IV** | | |
| --- | --- | --- | --- | --- | --- | --- | --- | --- | --- |
|  | **Estimate ± SE** | **95% CI** | **p** | **Estimate ± SE** | **95% CI** | **p** | **Estimate ± SE** | **95% CI** | **p** |
| Constant (Control) | 88.3 ± 8.2 | [72.2, 104.4] | 0.632 | 88.3 ± 8.6 | [71.4, 105.2] | 0.452 | 82.8 ± 8.8 | [65.5, 100.0] | 0.77 |
| Baseline treatment  (*IV vs Control or IT vs Control or IT vs IV*) | -5.6 ± 11.6 | [-28.3, 17.2] |  | -9.2 ± 12.2 | [-33.1, 14.8] |  | -3.6 ± 12.4 | [-28.0, 20.7] |  |
| Time point # Treatment group |  |  |  |  |  |  |  |  |  |
| 3 months # UC-MSC group | 2.6 ± 5.3 | [-7.8, 12.9] | 0.628 | 0.5 ± 5.3 | [-9.9, 10.8] | 0.925 | -2.1 ± 3.7 | [-9.4, 5.3] | 0.581 |
| 6 months # UC-MSC group | 5.0 ± 5.3 | [-5.4, 15.4] | 0.344 | 2.0 ± 5.3 | [-8.3, 12.3] | 0.706 | -3.0 ± 3.7 | [-10.3, 4.3] | 0.422 |
| 12 months # UC-MSC group | 10.1 ± 5.3 | [-0.2, 20.5] | 0.055 | 11.4 ± 5.3 | [1.0, 21.8] | 0.032 | 1.3 ± 3.7 | [-6.1, 8.6] | 0.738 |

******* *Note: UC-MSCs = Umbilical cord-derived mesenchymal stem cells; FMS = Fine Motor Skills; IV = Intravenous; IT = Intrathecal. 'Constant' represents the baseline FMS left-hand score. 'Baseline treatment' indicates the estimated difference in baseline FMS left-hand scores between groups (IV vs Control, IT vs Control, IT vs IV). 'Time point × treatment group' represents the estimated change in FMS left-hand scores at 3, 6, and 12 months for each treatment group*

**Table S2C.** **Mixed-effects model analysis of FMS left-hand scores in patients with bilateral MCA involvement**

| **Model Parameters** | **IV vs control** | | | **IT vs control** | | | **IT vs IV** | | |
| --- | --- | --- | --- | --- | --- | --- | --- | --- | --- |
|  | **Estimate ± SE** | **95% CI** | **p** | **Estimate ± SE** | **95% CI** | **p** | **Estimate ± SE** | **95% CI** | **p** |
| Constant | 74.0 ± 16.4 | [41.9, 106.1] | 0.531 | 74.0 ± 18.7 | [37.4, 110.6] | 0.389 | 88.5 ± 13.7 | [61.6, 115.4] | 0.483 |
| Baseline treatment  (*IV vs Control or IT vs Control or IT vs IV*) | 14.5 ± 23.2 | [-30.9, 59.9] |  | 36.0 ± 41.8 | [-45.8, 117.8] |  | 21.5 ± 30.7 | [-38.6, 81.6] |  |
| Time point # Treatment group |  |  |  |  |  |  |  |  |  |
| 3 months # UC-MSC group | 12.5 ± 15.8 | [-18.5, 43.5] | 0.43 | 8.8 ± 26.6 | [-43.4, 60.9] | 0.742 | -3.8 ± 23.3 | [-49.4, 41.9] | 0.872 |
| 6 months # UC-MSC group | 23.3 ± 15.8 | [-7.8, 54.3] | 0.142 | 10.3 ± 26.6 | [-41.9, 62.4] | 0.700 | -13 ± 23.3 | [-58.7, 32.7] | 0.577 |
| 12 months # UC-MSC group | 14.8 ± 15.8 | [-16.3, 45.8] | 0.351 | 6.8 ± 26.6 | [-45.4, 58.9] | 0.800 | -8 ± 23.3 | [-53.7, 37.7] | 0.731 |

******* *Note: UC-MSCs = Umbilical cord-derived mesenchymal stem cells; FMS =* *Fine Motor Skills; IV = Intravenous; IT = Intrathecal**; MCA=Middle Cerebral Artery. 'Constant' represents the baseline FMS left-hand score. 'Baseline treatment' indicates the estimated difference in baseline FMS left-hand scores between groups (IV vs Control, IT vs Control, IT vs IV). 'Time point × treatment group' represents the estimated change in FMS left-hand scores at 3, 6, and 12 months for each treatment group*


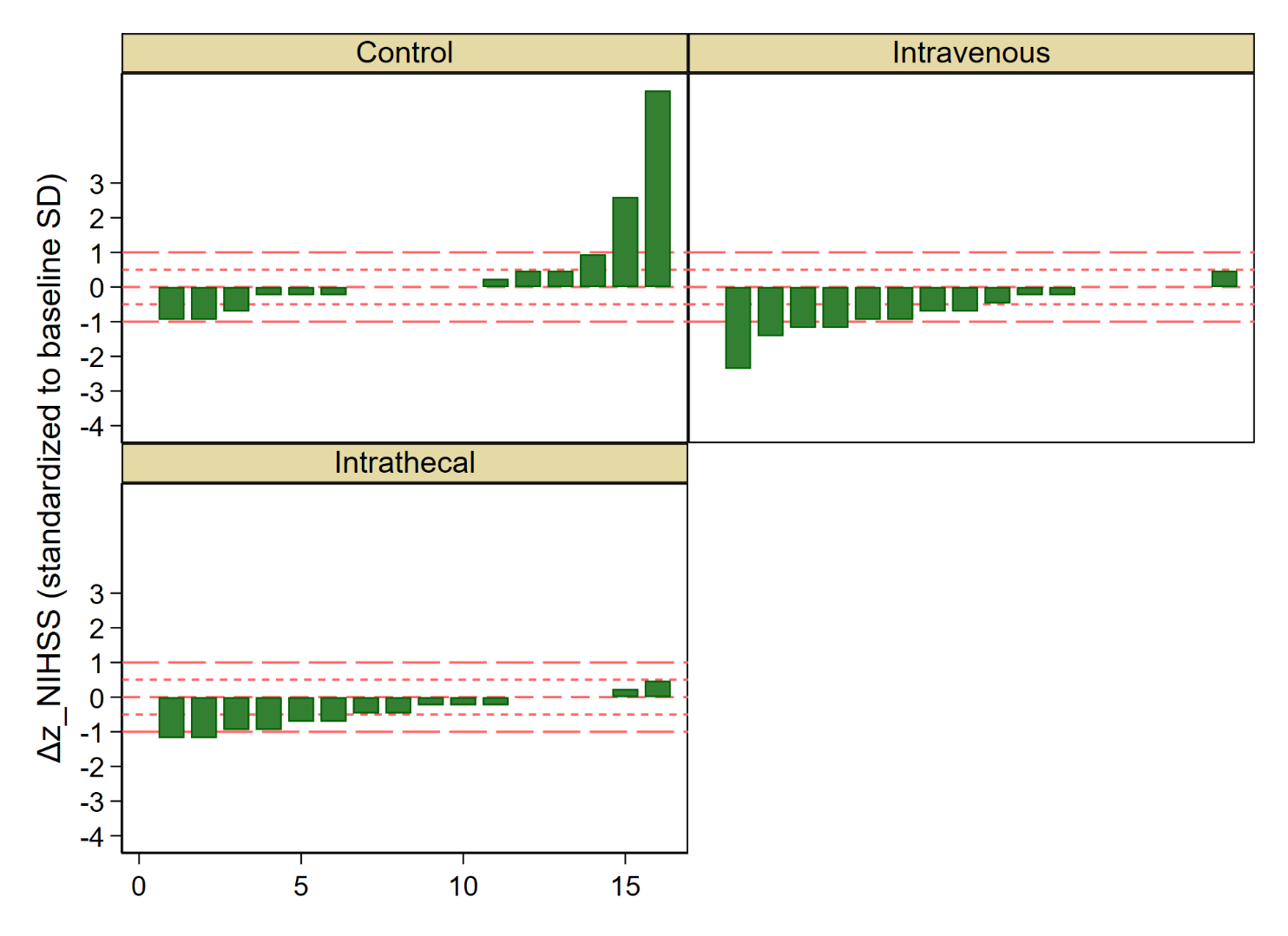


**Figure S3. Rank‑ordered patient change in NIHSS at 12 months (Δz standardized to baseline SD) between groups**

*Figure legend*: Each bar represents one participant ordered by Δz_NIHSS, downward bars indicate improvement defined as a lower neurological deficit versus baseline using the group’s baseline SD, dashed lines denote 0.5 SD and 1.0 SD reference thresholds, panels display Control, Intravenous infusion, and Intrathecal infusion at 12 months for visual comparison. Participants with no change from baseline (Δz = 0) are not visible on the plot.


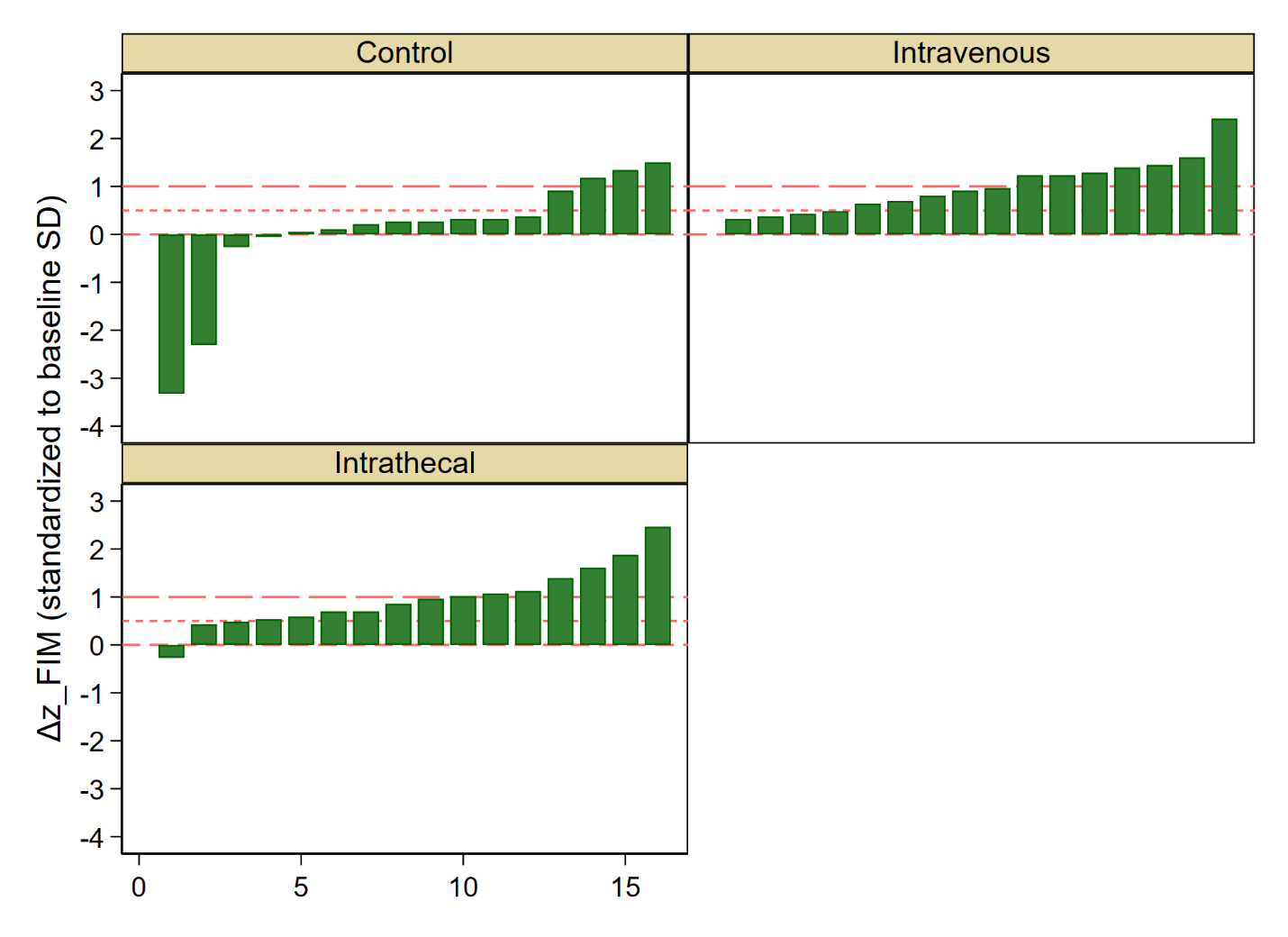


**Figure S4. Rank‑ordered patient change in FIM total at 12 months (Δz standardized to baseline SD) between groups**

*Figure legend*: Each bar represents one participant ranked by Δz_FIM total, upward bars indicate improvement defined as greater functional independence in activities of daily living, dashed lines denote 0.5 SD and 1.0 SD reference thresholds, panels display Control, Intravenous infusion, and Intrathecal infusion at 12 months.


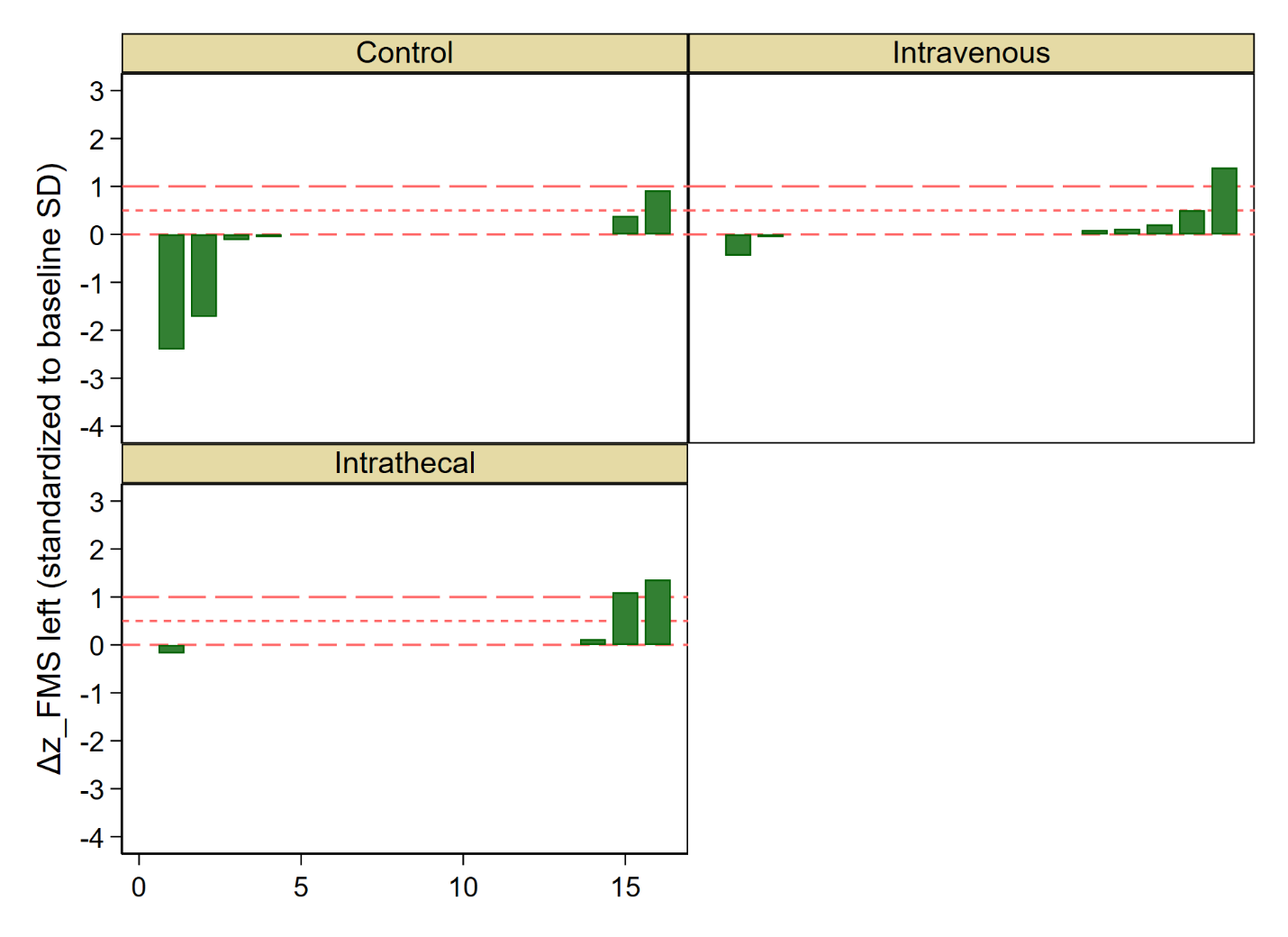


**Figure S5. Rank‑ordered patient change in FMS left at 12 months (Δz standardized to baseline SD) between groups**

*Figure legend*: Each bar represents one participant ordered by Δz_FMS left, upward bars indicate improvement defined as better left‑hand dexterity versus baseline, dashed lines denote 0.5 SD and 1.0 SD reference thresholds, panels display Control, Intravenous, and Intrathecal at 12 months. Participants with no change from baseline (Δz = 0) are not visible on the plot.


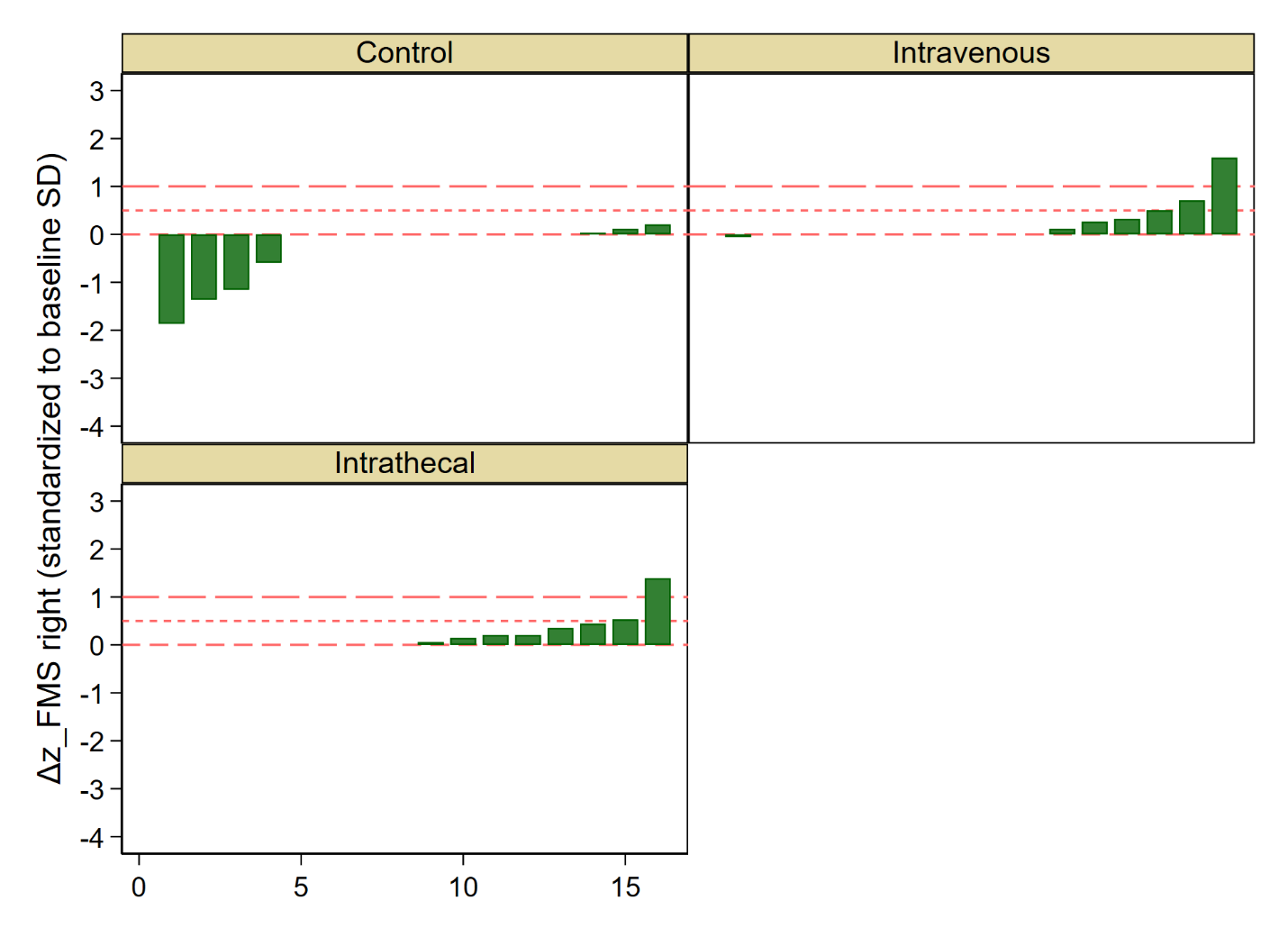


**Figure S6. Rank‑ordered patient change in FMS right at 12 months (Δz standardized to baseline SD) between groups**

*Figure legend*: Each bar is one participant ordered by Δz_FMS right, upward bars indicate improvement defined as better right‑hand dexterity versus baseline, dashed lines mark 0.5 SD and 1.0 SD, panels show Control, intravenous, and intrathecal at 12 months. Participants with no change from baseline (Δz = 0) are not visible on the plot.


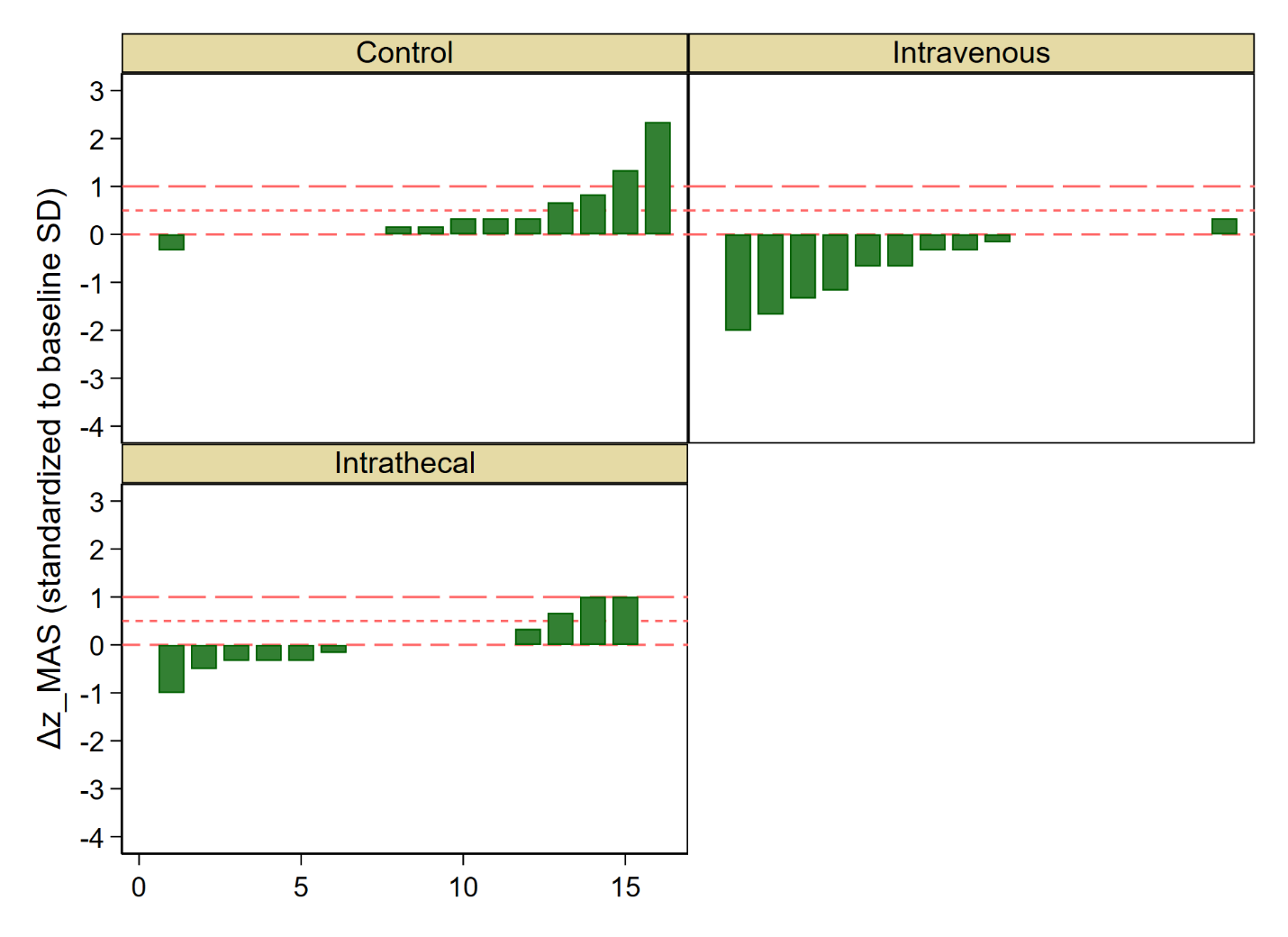


**Figure S7. Rank‑ordered patient change in MAS at 12 months (Δz standardized to baseline SD) between groups**

*Figure legend*: Each bar represents one participant ordered by Δz_MAS, downward bars indicate improvement defined as less spasticity versus baseline, dashed lines denote 0.5 SD and 1.0 SD reference thresholds, panels display Control, Intravenous, and Intrathecal at 12 months. Participants with no change from baseline (Δz = 0) are not visible on the plot.


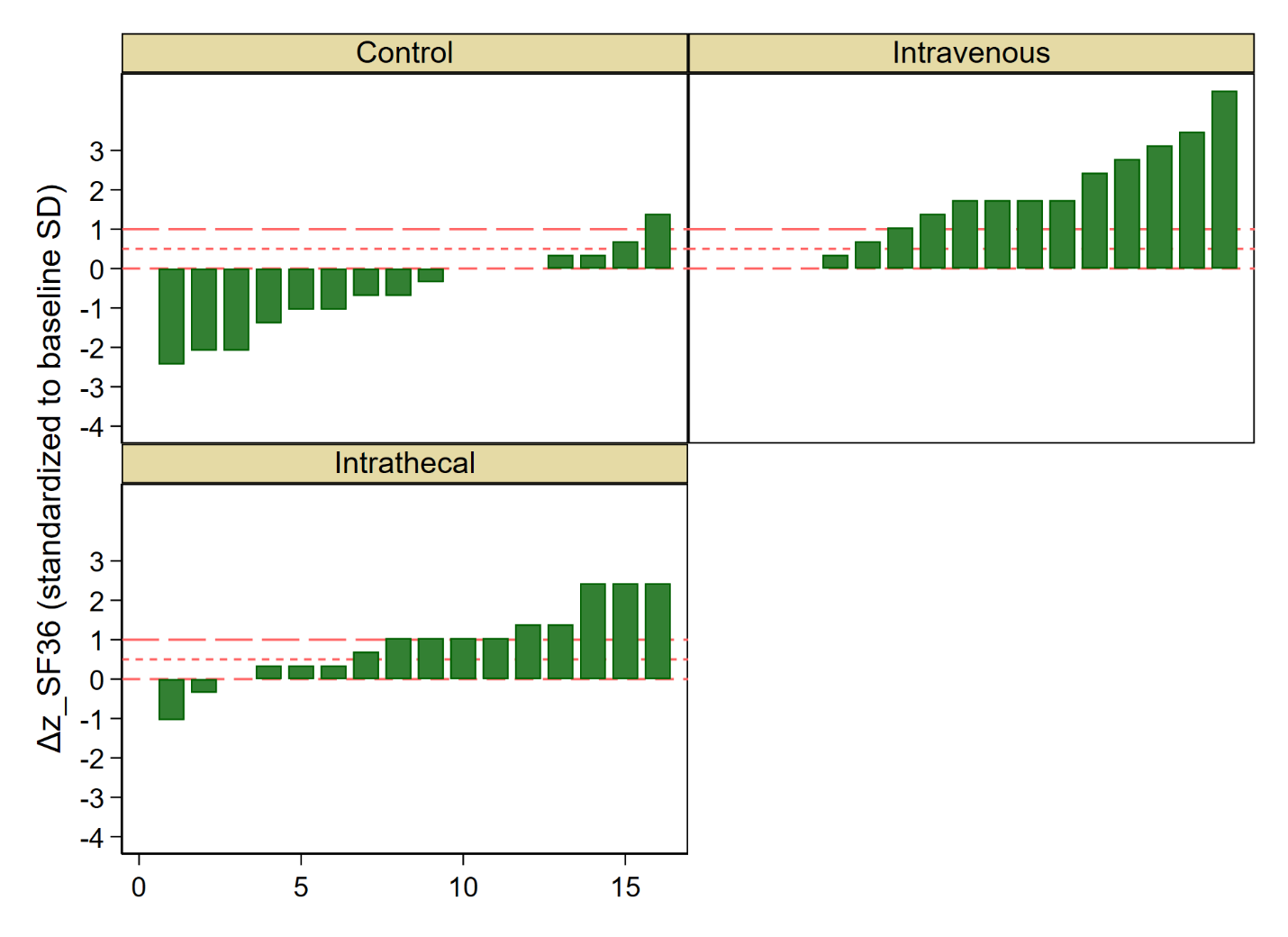


**Figure S8. Rank‑ordered patient‑level change in SF‑36 at 12 months (Δz standardized to baseline SD) between groups**

*Figure legend*: Each bar represents one participant ordered by Δz_SF36 upward bars indicate improvement defined as higher health‑related quality of life versus baseline using the group’s baseline SD dashed lines denote 0.5 SD and 1.0 SD reference levels panels display Control IV and IT side‑by‑side for visual comparison at 12 months. Participants with no change from baseline (Δz = 0) are not visible on the plot.

**Table S3. Detailed analysis of MRI improvement distribution**

**by treatment groups and time points**

| **Time point** | **IV**  **(n=16)** | **IT**  **(n=16)** | **Control**  **(n=16)** | **Chi-square** | ***p*** |
| --- | --- | --- | --- | --- | --- |
| 6 months | 3 (18.8%) | 2 (12.5%) | 2 (12.5%) | 0.3345 | 0.846 |
| 12 months | 4 (25%) | 2 (12.5%) | 3 (18.8%) | 0.8205 | 0.663 |

******Note: IV=Intravenous; IT= Intrathecal; values are presented as N (%)*
