# Supplementary material for: Intrathecal versus intravenous umbilical cord mesenchymal stem cells for ischemic stroke sequelae
Source: Stem Cells Transl Med. 2025 Nov 24;14(12):szaf063. doi: 10.1093/stcltm/szaf063 (PMC12641229; doi:10.1093/stcltm/szaf063)
Supplement: szaf063_Supplementary_Data [file szaf063_supplementary_data.zip › Table S1.docx]

**Table S1. Lesion locations at baseline in each group**

| **Lesion location** | **IV**  **(n=16)** | **IT**  **(n=16)** | **Control**  **(n=16)** |
| --- | --- | --- | --- |
| Middle cerebral artery bilateral | 6 (37.5%) | 1 (6.3 %) | 4 (25.0%) |
| Middle cerebral artery - right | 3 (18.8%) | 5 (31.3 %) | 5 (31.3%) |
| Middle cerebral artery - left | 5 (31.3%) | 8 (50.0 %) | 7 (43.8%) |
| Bilateral anterior cerebral artery | 1 (6.3%) | 1 (6.3%) | 0 (0%) |
| Left + anterior cerebral artery | 1 (6.3%) | 1 (6.3%) | 0 (0%) |

**Note: IV = Intravenous; IT= Intrathecal; The data are presented as N (%)*
