# Supplementary material for: Intrathecal versus intravenous umbilical cord mesenchymal stem cells for ischemic stroke sequelae
Source: Stem Cells Transl Med. 2025 Nov 24;14(12):szaf063. doi: 10.1093/stcltm/szaf063 (PMC12641229; doi:10.1093/stcltm/szaf063)
Supplement: szaf063_Supplementary_Data [file szaf063_supplementary_data.zip › Table S2A.docx]

**Table S2A.** **Comparison of FMS right-hand scores over time between groups via a mixed-effects model**

| **Model Parameters** | **IV vs control** | | | **IT vs control** | | | **IT vs IV** | | |
| --- | --- | --- | --- | --- | --- | --- | --- | --- | --- |
|  | **Estimate ± SE** | **95% CI** | **p** | **Estimate ± SE** | **95% CI** | **p** | **Estimate ± SE** | **95% CI** | **p** |
| Constant | 75.3 ± 8.6 | [58.5, 92.0] | 0.967 | 75.3 ± 8.8 | [58.0, 92.5] | 0.372 | 74.8 ± 8.6 | [57.9, 91.6] | 0.382 |
| Baseline treatment  (*IV vs Control or IT vs Control or IT vs IV*) | -0.5 ± 12.1 | [-24.2, 23.2] |  | -11.1 ± 12.5 | [-35.5, 13.3] |  | -10.6 ± 12.2 | [-34.5, 13.2] |  |
| Time point # Treatment group |  |  |  |  |  |  |  |  |  |
| 3 months # UC-MSC group | 6.3 ± 4.2 | [-2.0, 14.5] | 0.139 | 3.4 ± 4.6 | [-5.7, 12.4] | 0.465 | -2.9 ± 4.2 | [-11.2, 5.4] | 0.497 |
| 6 months # UC-MSC group | 13.3 ± 4.2 | [5.0, 21.5] | 0.002 | 8.4 ± 4.6 | [-0.6, 17.5] | 0.068 | -4.8 ± 4.2 | [-13.1, 3.5] | 0.255 |
| 12 months # UC-MSC group | 17.1 ± 4.2 | [8.8, 25.3] | <0.001 | 16.8 ± 4.6 | [7.8, 25.9] | <0.001 | -0.3 ± 4.2 | [-8.5, 8.0] | 0.953 |

******* *Note: UC-MSCs = Umbilical cord-derived mesenchymal stem cells; FMS =* *Fine Motor Skills; IV = Intravenous; IT = Intrathecal. 'Constant' represents the baseline FMS right-hand score. 'Baseline treatment' indicates the estimated difference in baseline FMS right-hand scores between groups (IV vs Control, IT vs Control, IT vs IV). 'Time point × treatment group' represents the estimated change in FMS right-hand scores at 3, 6, and 12 months for each treatment group*
