# Supplementary material for: Intrathecal versus intravenous umbilical cord mesenchymal stem cells for ischemic stroke sequelae
Source: Stem Cells Transl Med. 2025 Nov 24;14(12):szaf063. doi: 10.1093/stcltm/szaf063 (PMC12641229; doi:10.1093/stcltm/szaf063)
Supplement: szaf063_Supplementary_Data [file szaf063_supplementary_data.zip › Table S2B.docx]

**Table S2B.** **Comparison of FMS changes in left-hand pain scores over time among the UC-MSC infusion groups via either the IV or IT route and the control group via the mixed-effects model**

| **Model Parameters** | **IV vs control** | | | **IT vs control** | | | **IT vs IV** | | |
| --- | --- | --- | --- | --- | --- | --- | --- | --- | --- |
|  | **Estimate ± SE** | **95% CI** | **p** | **Estimate ± SE** | **95% CI** | **p** | **Estimate ± SE** | **95% CI** | **p** |
| Constant (Control) | 88.3 ± 8.2 | [72.2, 104.4] | 0.632 | 88.3 ± 8.6 | [71.4, 105.2] | 0.452 | 82.8 ± 8.8 | [65.5, 100.0] | 0.77 |
| Baseline treatment  (*IV vs Control or IT vs Control or IT vs IV*) | -5.6 ± 11.6 | [-28.3, 17.2] |  | -9.2 ± 12.2 | [-33.1, 14.8] |  | -3.6 ± 12.4 | [-28.0, 20.7] |  |
| Time point # Treatment group |  |  |  |  |  |  |  |  |  |
| 3 months # UC-MSC group | 2.6 ± 5.3 | [-7.8, 12.9] | 0.628 | 0.5 ± 5.3 | [-9.9, 10.8] | 0.925 | -2.1 ± 3.7 | [-9.4, 5.3] | 0.581 |
| 6 months # UC-MSC group | 5.0 ± 5.3 | [-5.4, 15.4] | 0.344 | 2.0 ± 5.3 | [-8.3, 12.3] | 0.706 | -3.0 ± 3.7 | [-10.3, 4.3] | 0.422 |
| 12 months # UC-MSC group | 10.1 ± 5.3 | [-0.2, 20.5] | 0.055 | 11.4 ± 5.3 | [1.0, 21.8] | 0.032 | 1.3 ± 3.7 | [-6.1, 8.6] | 0.738 |

******* *Note: UC-MSCs = Umbilical cord-derived mesenchymal stem cells; FMS = Fine Motor Skills; IV = Intravenous; IT = Intrathecal. 'Constant' represents the baseline FMS left-hand score. 'Baseline treatment' indicates the estimated difference in baseline FMS left-hand scores between groups (IV vs Control, IT vs Control, IT vs IV). 'Time point × treatment group' represents the estimated change in FMS left-hand scores at 3, 6, and 12 months for each treatment group*
