# Supplementary material for: Intrathecal versus intravenous umbilical cord mesenchymal stem cells for ischemic stroke sequelae
Source: Stem Cells Transl Med. 2025 Nov 24;14(12):szaf063. doi: 10.1093/stcltm/szaf063 (PMC12641229; doi:10.1093/stcltm/szaf063)
Supplement: szaf063_Supplementary_Data [file szaf063_supplementary_data.zip › Table S2C.docx]

**Table S2C.** **Mixed-effects model analysis of FMS left-hand scores in patients with bilateral MCA involvement**

| **Model Parameters** | **IV vs control** | | | **IT vs control** | | | **IT vs IV** | | |
| --- | --- | --- | --- | --- | --- | --- | --- | --- | --- |
|  | **Estimate ± SE** | **95% CI** | **p** | **Estimate ± SE** | **95% CI** | **p** | **Estimate ± SE** | **95% CI** | **p** |
| Constant | 74.0 ± 16.4 | [41.9, 106.1] | 0.531 | 74.0 ± 18.7 | [37.4, 110.6] | 0.389 | 88.5 ± 13.7 | [61.6, 115.4] | 0.483 |
| Baseline treatment  (*IV vs Control or IT vs Control or IT vs IV*) | 14.5 ± 23.2 | [-30.9, 59.9] |  | 36.0 ± 41.8 | [-45.8, 117.8] |  | 21.5 ± 30.7 | [-38.6, 81.6] |  |
| Time point # Treatment group |  |  |  |  |  |  |  |  |  |
| 3 months # UC-MSC group | 12.5 ± 15.8 | [-18.5, 43.5] | 0.43 | 8.8 ± 26.6 | [-43.4, 60.9] | 0.742 | -3.8 ± 23.3 | [-49.4, 41.9] | 0.872 |
| 6 months # UC-MSC group | 23.3 ± 15.8 | [-7.8, 54.3] | 0.142 | 10.3 ± 26.6 | [-41.9, 62.4] | 0.700 | -13 ± 23.3 | [-58.7, 32.7] | 0.577 |
| 12 months # UC-MSC group | 14.8 ± 15.8 | [-16.3, 45.8] | 0.351 | 6.8 ± 26.6 | [-45.4, 58.9] | 0.800 | -8 ± 23.3 | [-53.7, 37.7] | 0.731 |

******* *Note: UC-MSCs = Umbilical cord-derived mesenchymal stem cells; FMS =* *Fine Motor Skills; IV = Intravenous; IT = Intrathecal**; MCA=Middle Cerebral Artery. 'Constant' represents the baseline FMS left-hand score. 'Baseline treatment' indicates the estimated difference in baseline FMS left-hand scores between groups (IV vs Control, IT vs Control, IT vs IV). 'Time point × treatment group' represents the estimated change in FMS left-hand scores at 3, 6, and 12 months for each treatment group*
