# Supplementary material for: Intrathecal versus intravenous umbilical cord mesenchymal stem cells for ischemic stroke sequelae
Source: Stem Cells Transl Med. 2025 Nov 24;14(12):szaf063. doi: 10.1093/stcltm/szaf063 (PMC12641229; doi:10.1093/stcltm/szaf063)
Supplement: szaf063_Supplementary_Data [file szaf063_supplementary_data.zip › Table S3.docx]

**Table S3. Detailed analysis of MRI improvement distribution**

**by treatment groups and time points**

| **Time point** | **IV**  **(n=16)** | **IT**  **(n=16)** | **Control**  **(n=16)** | **Chi-square** | ***p*** |
| --- | --- | --- | --- | --- | --- |
| 6 months | 3 (18.8%) | 2 (12.5%) | 2 (12.5%) | 0.3345 | 0.846 |
| 12 months | 4 (25%) | 2 (12.5%) | 3 (18.8%) | 0.8205 | 0.663 |

******Note: IV=Intravenous; IT= Intrathecal; values are presented as N (%)*
